# Steps for *Shigella* Gatekeeper Protein MxiC Function in Hierarchical Type III Secretion Regulation*[Fn FN2]

**DOI:** 10.1074/jbc.M116.746826

**Published:** 2016-12-14

**Authors:** A. Dorothea Roehrich, Enrica Bordignon, Selma Mode, Da-Kang Shen, Xia Liu, Maria Pain, Isabel Murillo, Isabel Martinez-Argudo, Richard B. Sessions, Ariel J. Blocker

**Affiliations:** From the ‡ School of Cellular and Molecular Medicine and School of Biochemistry, Medical Sciences Building, Faculty of Medical and Veterinary Sciences, University of Bristol, Bristol BS8 1TD, United Kingdom,; the § Fachbereich Physik, Freie Universität Berlin, 14195 Berlin, Germany, and; the ¶ Área de Genética, Facultad de Ciencias Ambientales y Bioquímica, Universidad de Castilla-La Mancha, E-45071 Toledo, Spain

**Keywords:** biophysics, cellular regulation, electron paramagnetic resonance (EPR), Gram-negative bacteria, molecular genetics, protein secretion, type III secretion system (T3SS), MxiC, Shigella

## Abstract

Type III secretion systems are complex nanomachines used for injection of proteins from Gram-negative bacteria into eukaryotic cells. Although they are assembled when the environmental conditions are appropriate, they only start secreting upon contact with a host cell. Secretion is hierarchical. First, the pore-forming translocators are released. Second, effector proteins are injected. Hierarchy between these protein classes is mediated by a conserved gatekeeper protein, MxiC, in *Shigella*. As its molecular mechanism of action is still poorly understood, we used its structure to guide site-directed mutagenesis and to dissect its function. We identified mutants predominantly affecting all known features of MxiC regulation as follows: secretion of translocators, MxiC and/or effectors. Using molecular genetics, we then mapped at which point in the regulatory cascade the mutants were affected. Analysis of some of these mutants led us to a set of electron paramagnetic resonance experiments that provide evidence that MxiC interacts directly with IpaD. We suggest how this interaction regulates a switch in its conformation that is key to its functions.

## Introduction

Type III secretion systems (T3SSs)^5^ are central devices in the virulence of many major Gram-negative bacterial pathogens of humans, animals, and plants. They translocate virulence proteins into the membranes and cytoplasm of eukaryotic host cells to manipulate them during infection. T3SSs are key to the virulence of enteric pathogens such as *Escherichia coli*, *Salmonella*, and *Shigella* species.

*Shigella* species are the etiological agent of bacillary dysentery in humans (1). The *Shigella* T3SS consists of a cytoplasmic portion and a transmembrane region traversing both bacterial membranes, into which a hollow needle, made of MxiH, is embedded protruding from the bacterial surface (2). Physical contact with eukaryotic host cells activates the secretion system, which initiates secretion and leads to creation of a pore, formed by the bacterial proteins IpaB and IpaC, in host cell membranes (3). The effectors are translocated through the needle (4) and pore channels to facilitate host cell invasion (3). The needle tip complex (TC), which contains IpaD and IpaB, is the host cell sensor and transforms itself into the translocation pore (5) via addition of IpaC upon secretion activation (6, 7). IpaD is hydrophilic and required for tip recruitment of the other two proteins, which are hydrophobic, and hence chaperoned by IpgC intrabacterially (8). The three proteins are collectively called the translocators.

T3SSs are assembled, using a broadly conserved morphogenesis pathway (9), following detection of environmental cues indicating entry into the host. In addition, virulence effectors acting late in the host cell manipulation cascade are only expressed once the presynthesized early effectors have been secreted at host cell contact. Most components and/or molecular mechanisms of these regulatory pathways diverge from one T3SS-carrying organism to another (10). However, one regulatory cascade is conserved, a process allowing hierarchical secretion of substrates, although the stages it covers vary as follows: needle *versus* translocator components in plant pathogens or translocators and then early effectors in animal ones (11).

Here, we focus on how this cascade functions in animal pathogens. After T3SS assembly, effector secretion is prevented through the concerted action of surface TC proteins and regulators that control secretion from within the bacterial cytoplasm. The TC may prevent premature effector secretion by allosterically constraining the T3SS in a secretion “off” conformation without blocking the secretion channel (12–14). Upon physical contact of the TC with host cells, a signal, termed Signal 1, is transmitted via the TC (15) and needle (12, 16) to the cytoplasm where it triggers secretion. Next, translocators are secreted to form the pore in the host cell membrane (3). Successful pore formation at the needle tip generates Signal 2, also transmitted via the needle, that allows inactivation or T3S-mediated removal of a conserved cytoplasmic regulatory protein MxiC in *Shigella* (12, 16). Then the early effector proteins are secreted and translocated into the host cell, and late effector expression is activated (17).

MxiC belongs to a class of “gatekeeper” proteins that is conserved among different type III secretion systems (18). They repress effector secretion in the absence of a secretion signal but have different roles in translocator secretion, impairing it in a Δ*mxiC* mutant (12) while stimulating it in a *Yersinia* Δ*yopN* mutant (19). Although gatekeepers are clearly involved in the cytoplasmic steps controlling T3SS secretion hierarchy upon activation, their mechanism of action remains unclear.

The gatekeepers have conserved structures (20, 21). After an N-terminal secretion signal and putative chaperone-binding domain (CBD), three α-helical X-bundles (domains 1–3; see supplemental Fig. S1, *A–C*) form a flat, elongated structure (21) typical for “hub proteins” regulating processes via interaction with multiple partners. In some species, gatekeepers are composed of two proteins where the second polypeptide covers the C-terminal X-bundle (domain 3; supplemental Fig. S1, *D* and *E*) (20).

MxiC is secreted by the type III secretion system (22). Its N-terminal 30 residues contain the secretion signal (23). Immediately thereafter is a domain similar to the chaperone-binding domain of *Yersinia* YopN (20, 21). This domain is partially conserved (18) even though not every MxiC homolog has an identified chaperone. Although this area is enriched in hydrophobic residues that mediate interactions with the chaperones, fewer hydrophobic residues are found in MxiC.

Many type III secreted proteins are bound by a chaperone inside the bacterium. These chaperones have various roles, including stabilization of their binding partners, aiding their secretion and mediation of secretion hierarchy (24). Several MxiC homologs bind to specific heterodimeric chaperones. For instance, *Yersinia* YopN binds to the SycN/YscB heterodimer (20, 25). It wraps around its heterodimeric chaperone in a conformation similar to other effector-chaperone complexes (20). This domain is disordered in the absence of the chaperones. Interestingly, the first ∼75 residues of MxiC are also likely to be disordered (21). Yet, so far no chaperone has been identified for MxiC (23).

MxiC's helix 9 of is a straight helix, although the structurally equivalent helix in YopN/TyeA is kinked into two smaller helices. The structure of the EPEC MxiC homolog, SepL, is also bent at an equivalent location (26). Thus, one face of the molecule is flat in MxiC, although it is concave in YopN/TyeA and SepL (21, 26). Interestingly, this surface contains a negatively charged patch (Glu-201, Glu-276, and Glu-293 (21)) that we showed is important for MxiC functions that involve IpaD (15). Furthermore, the *Chlamydia* hydrophobic translocator chaperone Scc3 binds to its gatekeeper at the flat interface between domains 2 and 3 (27), which the kink in the YopN/TyeA renders convex. Deane *et al.* (21) already suggested this structural difference between MxiC and YopN/TyeA could be a “conformational switch,” and these new findings suggest it might allow the switch from hydrophilic to hydrophobic translocator secretion.

**FIGURE 1.**
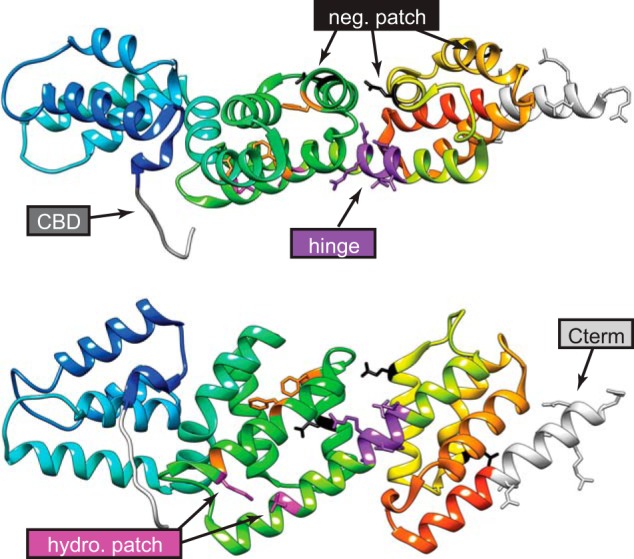
**Location of MxiC mutations used in this work.** *Top*, MxiC structure 2VJ4 (chain A) colored from *blue* at the N terminus, where MxiC Cys-74 is the first residue in the first crystallized helix, to *red* at the C terminus (*Cterm*). Therefore, the N terminus and most of CBD are not shown. *Bottom*, same as MxiC rotated by 90° about its long axis. Mutated residues are colored according to their design group, as labeled on the figure. *Orange* indicates mutations in the hydrophobic core of the protein, made by others (19, 33), which lead to loss of function. *Neg*, negative; *hydro*, hydrophobic.

To dissect MxiC's interconnected functions, we used site-directed mutagenesis. Mutant design was guided by the description of MxiC structure by Deane *et al.* (21) and the sequence alignment of MxiC homologs by Pallen *et al.* (18). Our mutations (Fig. 1) focused on the N-terminal non-crystallized region and domains 2 and 3 of the crystal structure (21). We identified mutants predominantly affecting all known features of MxiC regulation as follows: secretion of translocators, MxiC, and effectors. Using molecular genetics to map at which point in the regulatory cascade the mutants were affected, we further dissected MxiC's role. Analysis of some of these mutants led us to electron paramagnetic resonance (EPR) experiments that, together with phenotypic analysis of the mutants, provide evidence that MxiC's conformation is regulated via a direct interaction with IpaD.

## Results

### 

#### 

##### MxiC's Secretion Signal Is Not Required for Promoting Inducible Translocator Secretion

A non-secretable form of MxiC lacking residues 2–30 is unable to prevent effector secretion (23). However, MxiC also regulates inducible translocator secretion (12). This had not yet been characterized when the *mxiC*Δ*Nterm* mutant was first described. We thus investigated whether MxiC's two roles could be uncoupled.

**FIGURE 2.**
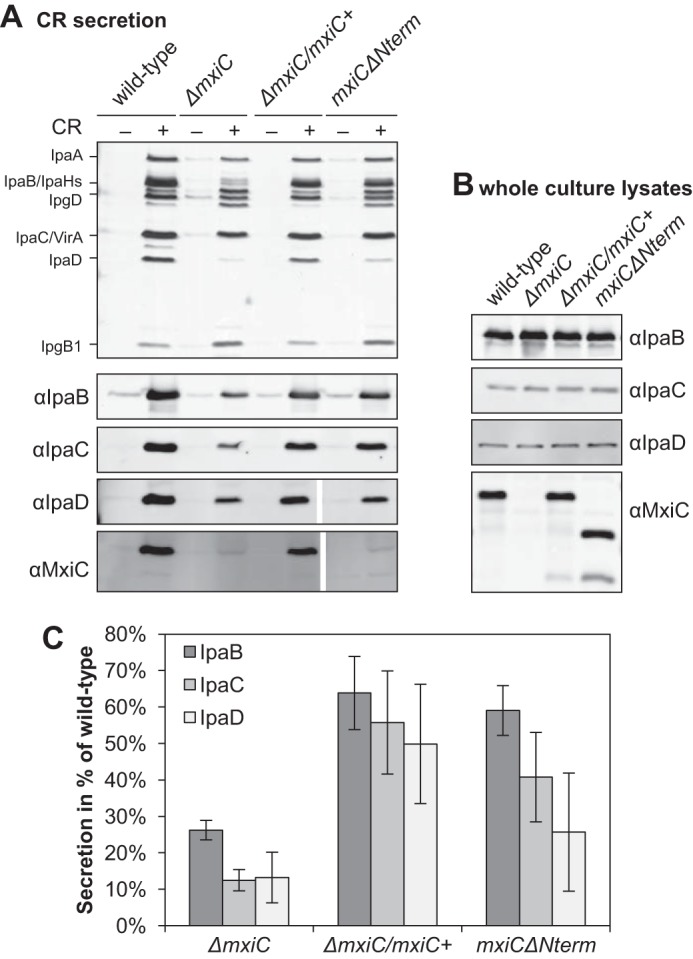
**MxiC without its secretion signal is still able to promote inducible translocator secretion.** *A*, protein secretion in response to the artificial inducer CR. Samples from *Shigella* wild type, Δ*mxiC* mutant, complemented strain (Δ*mxiC*/*mxiC*+, grown with 25 μm IPTG), and *mxiC*Δ*Nterm* (in the Δ*mxiC* background, grown with 100 μm IPTG) were collected as described under “Experimental Procedures,” silver-stained (*top panel*), and Western-blotted with the indicated antibodies (*bottom panels*). All samples probed with the same antibody were analyzed on the same gel. Results shown are representative of at least two independent experiments. *B*, total protein expression levels in whole culture lysates. Samples were collected as described under “Experimental Procedures” and Western-blotted with the indicated antibodies. *C*, quantification of translocator secretion after CR induction. Samples from two independent experiments, one of them performed in duplicate, were quantified on Western blottings. The averages and standard deviations of the wild-type normalized data are displayed. There is an overall difference between proteins and strains in an ANOVA (*p* < 0.01 and *p* ≤ 0.001, respectively). In pairwise comparisons, the difference between Δ*mxiC* and both the complemented strain and *mxiC*Δ*Nterm* is statistically significant (post hoc test with Bonferroni correction, *p* < 0.001 and *p* < 0.01, respectively), although the difference between complemented strain and *mxiC*Δ*Nterm* is not significant.

We generated an *mxiC*Δ*Nterm* mutant equivalent to that of Botteaux *et al.* (23). In our hands, MxiCΔNterm was unstable, whereas wild-type *mxiC* was induced to wild-type levels after addition of 25 μm IPTG, and *mxiC*Δ*Nterm* was only expressed at similar levels after addition of 100 μm IPTG. Furthermore, MxiCΔNterm seemed partially degraded (Fig. 2*B*).

As described previously, MxiCΔNterm is not secreted after Congo red (CR) induction (Fig. 2*A*) (23) and effector proteins are leaked (data not shown) (23). Furthermore, MxiCΔNterm was not secreted in a Δ*ipaB* constitutive secretor background (supplemental Fig. S2), indicating deletion of the N terminus affects its ability to become secreted. However, secretion of IpaB, IpaC, and IpaD was unaffected in *mxiC*Δ*Nterm*; small differences between Δ*mxiC*/*mxiC*+ and *mxiC*Δ*Nterm* were not statistically significant (Fig. 2, *A* and *C*). Thus, the ability of MxiC to prevent effector secretion and to promote inducible translocator secretion are not coupled, and only the former requires its secretion signal.

##### MxiC's C Terminus Is Essential

The 11 C-terminal residues of the EPEC MxiC homolog, SepL, are required for regulation of effector secretion but not for translocator secretion (28). As a complementary experiment, we deleted the last 14 residues of MxiC (Fig. 1, *bottom*), which are equivalent to the last 11 residues of SepL (supplemental Fig. S3) (18). The resulting *mxiC*Δ*Cterm* mutant was stably expressed but unable to complement Δ*mxiC* (supplemental Fig. S4); translocators were only weakly induced, and effector proteins were leaked. In addition, MxiCΔCterm itself was only poorly secreted (supplemental Fig. S4) even in Δ*ipaB* (supplemental Fig. S2). Therefore, unlike for SepL, MxiC's C terminus is essential.

##### Putative Chaperone-binding Domain of MxiC Regulates Its Secretion and Hence That of the Effectors

**FIGURE 3.**
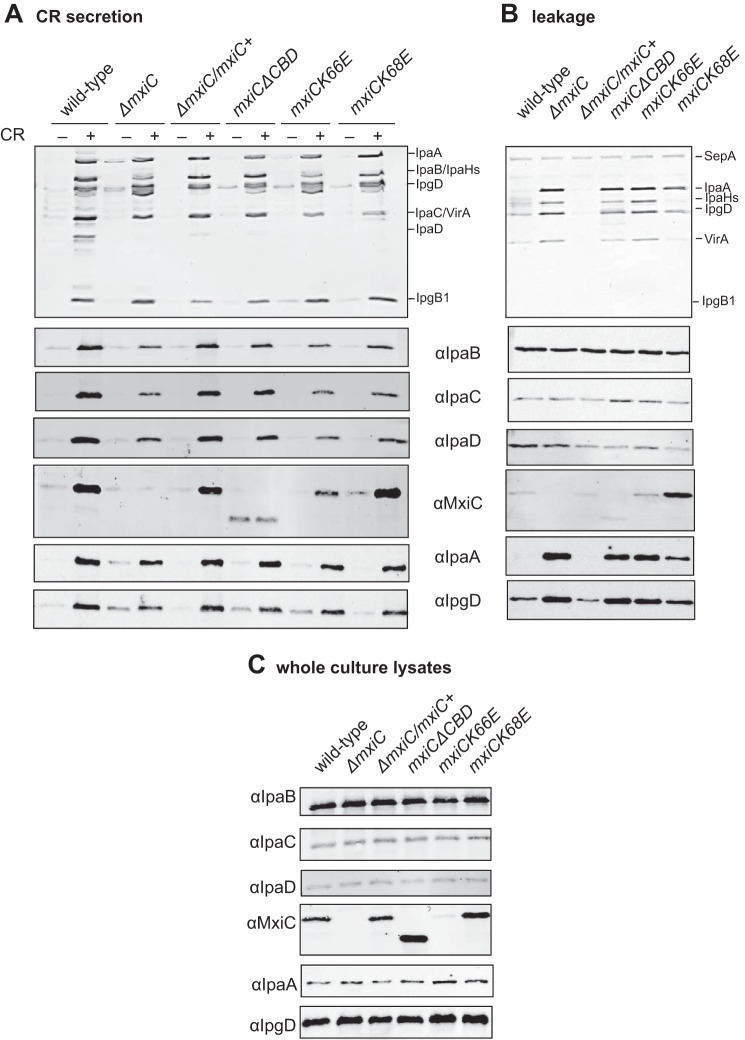
**Putative chaperone-binding domain regulates MxiC secretion and is required for blocking effector secretion.** *A*, protein secretion in response to the artificial inducer CR. Samples from the complemented strain (Δ*mxiC*/*mxiC*+) and indicated *mxiC* mutants (in the Δ*mxiC* background) were collected as described under “Experimental Procedures,” silver-stained (*top panel*), and Western-blotted with the indicated antibodies (*bottom panels*). *B*, exponential leakage. Samples were collected as described under “Experimental Procedures,” silver-stained (*top panel*), and Western-blotted with the indicated antibodies (*bottom panels*). *C*, total protein expression levels in whole culture lysates. Samples were collected as described under “Experimental Procedures” and Western-blotted with the indicated antibodies. Results shown are representative of at least two independent experiments.

The chaperone-binding domain of YopN has a chaperone-independent role in secretion regulation (29). We thus wondered whether MxiC's putative chaperone-binding domain is required for its function. First, we deleted the whole CBD (Fig. 1, *top*) to generate *mxiC*Δ*CBD*, lacking residues 32–72. This construct was stably expressed, but it was leaked and only weakly secreted upon CR induction (Fig. 3*A*). Interestingly, secretion of MxiCΔCBD was enhanced in a Δ*ipaB* background (supplemental Fig. S2). This indicates the chaperone-binding domain influences the regulation of MxiC secretion rather than the intrinsic ability of the protein to become secreted. Furthermore, although *mxiC*Δ*CBD* could not block effector secretion, it was able to promote translocator secretion (Fig. 3*A*).

Next, we investigated which YopN residues contacting the chaperones are conserved in MxiC. As the alignment of this area is ambiguous (supplemental Fig. S5), we made several single and combined charge-swap mutations. *mxiCK66E* was unable to complement Δ*mxiC*. Paradoxically, the mutant protein was detected in culture supernatants and, to a lesser degree, in the supernatants after CR induction, but only low levels were detected in whole culture lysates (Fig. 3*C*). *mxiC*(*D46K,D49K*) had a wild-type-like phenotype, except for some premature MxiC secretions. *mxiC*(*D46K,D49K,K66E*) leaked MxiC at high levels and was unable to prevent effector secretion (data not shown). A similar phenotype was observed in *mxiCK68E* (Fig. 3). Furthermore, *mxiCK68E* and *mxiCK66E*, but not *mxiC*Δ*CBD*, displayed reduced induction of translocator secretion, suggesting the effect of the point mutations on translocator secretion is indirect. Therefore, the putative chaperone-binding domain is required for regulating MxiC secretion and for preventing effector secretion.

MxiC lacking its secretion signal is not secreted and is also unable to prevent effector secretion (23). In an *mxiHK69A* mutant where MxiC is not secreted, effectors are also not released (12, 16). Thus, there is a correlation between “secretability” and the ability to block secretion. We hence tested whether MxiC needs to be quantitatively removed from the cytoplasm to release the block on effector secretion. We compared the levels of several secreted proteins in supernatants after induction with CR with levels in the corresponding total cultures, *i.e.* samples containing secreted and intracellular proteins. The translocators IpaB and IpaC were secreted nearly completely, whereas only 8 ± 6% of MxiC was secreted in the same period (supplemental Fig. S6). Translocator IpaD and early effector IpgD were secreted at intermediate levels of ∼40–50%. Thus, either only a small proportion of intracellular MxiC is involved in blocking secretion and/or its secretion *per se* is not required to release this block.

##### Any MxiC Chaperone Remains Unidentified

As MxiC's putative CBD is essential for regulating the protein's secretion and as *Yersinia* YopN needs its heterodimeric chaperone SycN/YscB for efficient secretion and regulation (25, 30), we wondered whether a chaperone is required for MxiC function. Three chaperones are encoded on the pWR100 virulence plasmid of *Shigella* that fall into the same general class as SycN and YscB: IpgA, IpgE, and Spa15 (31). Botteaux *et al.* (23) already found the lack of any of these proteins alone has no effect on MxiC secretion or stability. Thus, none of the *Shigella* class I chaperones work like SycN/YscB. However, their mechanism of action could be different, and thus we generated two double deletions (Δ*ipgE*Δ*spa15* and Δ*ipgE*Δ*ipgA*) and a triple deletion (Δ*ipgE*Δ*ipgA*Δ*spa15*). However, even the deletion of multiple chaperones had no effect on MxiC (supplemental Fig. S7). This suggests that none of these chaperones influences MxiC function. Therefore, either there are no such chaperones binding to the CBD of MxiC, or we have not identified them because of low sequence similarities. However, we think the latter is unlikely because MxiC is primarily monomeric in cytosol, and no known class 1 chaperone was identified, even using mass spectrometry after cross-linking (32).

##### Hydrophobic Patch on the Surface of MxiC Is Required for Blocking Effector Secretion

Deane *et al.* (21) noticed conservation of a patch of hydrophobic residues on the surface of MxiC's domain 2 (Fig. 1, *bottom*; Leu-222, Met-226, Gly-239, Leu-242, and Leu-245), suggesting it as a site for protein-protein interactions. Cherradi *et al.* (33) changed single residues in this patch to alanines. MxiC is not detectable in *mxiCL222A* and *mxiCL242A*, although three other mutations (M226A, G239A, and L245A) do not effect its function. We mutated residues Leu-222 and Leu-242 into serines and found that neither *mxiCL222S* and *mxiCL242S* nor the double mutant *mxiC*(*L222S,L242S*) had any effect on MxiC stability or function (data not shown).

**FIGURE 4.**
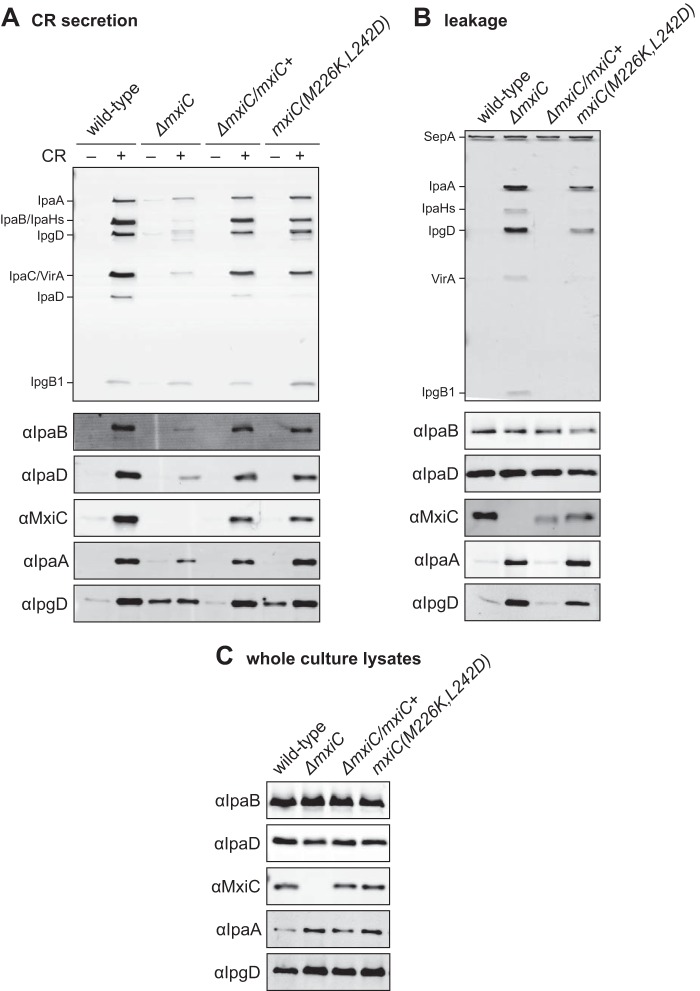
**Conserved hydrophobic patch on the surface of MxiC is involved in preventing effector secretion.** *A*, protein secretion in response to the artificial inducer CR. *Shigella* wild type, Δ*mxiC* mutant, complemented strain (Δ*mxiC*/*mxiC*+), and *mxiC*(*M226K,L242D*) (in the Δ*mxiC* background) were grown with 25 μm IPTG where required. Samples were collected as described under “Experimental Procedures,” silver-stained (*top panel*), and Western-blotted with the indicated antibodies (*bottom panels*). *B*, exponential leakage. Samples were collected as described under “Experimental Procedures,” silver-stained (*top panel*), and Western-blotted with the indicated antibodies (*bottom panels*). *C*, total protein expression levels in whole culture lysates. Samples were collected as described under “Experimental Procedures” and Western-blotted with the indicated antibodies. Results shown are representative of at least two independent experiments.

As change to a polar side chain may not have been enough to disrupt interactions in this area, we introduced two charges. The *mxiC*(*M226K,L242D*) mutant was stably expressed (Fig. 4*C*) and able to induce translocator secretion after induction (Fig. 4*A*). However, *mxiC*(*M226K,L242D*) was unable to prevent effector secretion (Fig. 4, *A* and *B*). Thus, the hydrophobic patch is involved in preventing effector secretion.

##### Mutations in a Putative “Hinge” Area of MxiC Lead to Altered Secretion Patterns

The sequence in MxiC helix 9, its putative “hinge” region (Fig. 1, *top*), is not conserved. However, it contains multiple serines, aspartates, valines, and threonines that are not classically helix favoring (34). Multiple secondary structure prediction programs suggest that even in MxiC, helix 9 would be broken (supplemental Fig. S8*B*). We generated a model of the “bent” wild-type protein using the YopN/TyeA structure (PDB code 1XL3 (20)) as template (supplemental Fig. S8*A*). In comparison with the crystal structure, the bent MxiC model is not only folded at the hinge region but undergoes a slight twisting motion. We then made three mutants as follows: one mutant introducing a proline (V256P; bent) that would likely cause a break in helix 9 and thus a conformation similar to that of YopN/TyeA; a second mutant introducing three glycines (T253G, S254G, and D255G; “wobble”) in the area to favor switching between both putative forms; and a third mutant that might stabilize a straight helix (I251A, T253A, S254A, and D255E; “straight”). MxiC Ile-251 is structurally equivalent to Phe-268 of YopN, which is sandwiched between hydrophobic residues in the core of TyeA, thus likely stabilizing the bent conformation.

**FIGURE 5.**
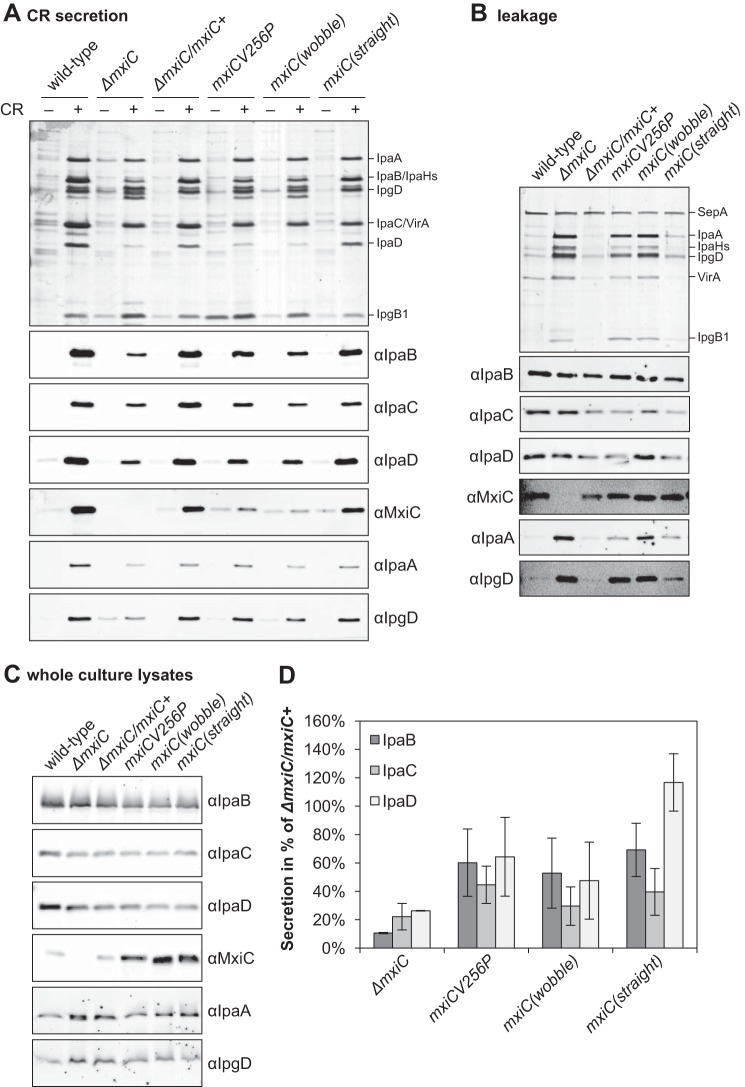
**Mutants designed to alter the conformation of MxiC show opposed phenotypes.** *A*, protein secretion in response to the artificial inducer CR. *Shigella* wild type, Δ*mxiC* mutant, complemented strain (Δ*mxiC*/*mxiC*+), and *mxiC* mutants (in the Δ*mxiC* background) were grown with 25 μm IPTG where required. Mutant *mxiC*(*T253G,S254G,D255G*) is abbreviated *mxiC*(*wobble*) and mutant *mxiC*(*I251A,T253A,S254A,D255E*) is abbreviated *mxiC*(*straight*). Samples were collected as described under “Experimental Procedures,” silver-stained (*top panel*), and Western-blotted with the indicated antibodies (*bottom panels*). *B*, exponential leakage. Samples were collected as described under “Experimental Procedures,” silver-stained (*top panel*), and Western-blotted with the indicated antibodies (*bottom panels*). *C*, total protein expression levels in whole culture lysates. Samples were collected as described under “Experimental Procedures” and Western-blotted with the indicated antibodies. *D*, quantification of translocator secretion after CR induction. Samples from three independent experiments were quantified on Western blottings and normalized against the complemented strain Δ*mxiC*/*mxiC*+. The averages and standard deviations are displayed. There is an overall difference between proteins and strains in an ANOVA (*p* < 0.01 and *p* < 0.001, respectively). In pairwise comparisons (post hoc test with Bonferroni correction), the difference between *mxiC*(*T253G,S254G,D255G*) and Δ*mxiC*/*mxiC*+ is statistically significant (*p* < 0.001), and the same is true for mutant *mxiCV256P*. Both strains are not significantly different from the Δ*mxiC* mutant. Mutant *mxiC*(*I251A,T253A,S254A,D255E*) is overall significantly different from Δ*mxiC* (*p* < 0.001), but not from the complemented strain. There are also significant differences between proteins in the *mxiC*(*I251A,T253A,S254A,D255E*) mutant (*p* < 0.01 in an ANOVA). Specifically, IpaD is significantly different from both IpaB and IpaC (post hoc test with Bonferroni correction, *p* < 0.05 and *p* < 0.01, respectively).

All mutant proteins were expressed at wild-type levels or better (Fig. 5*C*). The triple glycine mutant *mxiC*(*T253G,S254G,D255G*) behaved like Δ*mxiC*. MxiC was not secreted efficiently, and translocator secretion was reduced (43 ± 23% of the complemented strain), and effector proteins were leaked. Under the straight mutant *mxiC*(*I251A,T253A,S254A,D255E*), induced secretion of both IpaB and IpaC was reduced, whereas that of IpaD was as efficient as in the complemented strain or even increased (Fig. 5*D*). This mutant did not affect secretion of MxiC or effector proteins. The bent mutant *mxiCV256P* leaked high levels of a low molecular weight protein. We identified this band by mass spectrometry using a sample equivalent to the “−CR” sample in Fig. 5*A*. The top T3S-related protein identified was effector IpgB1 (6% of the total protein content). The similarly sized effector/anti-activator OspD1 was not detected in this band. The equivalent band from a Δ*mxiC* “+CR” sample was analyzed in parallel; again, IpgB1 was the top T3S-related hit (32). The *mxiCV256P* mutant also leaked MxiC at wild-type levels and other effector proteins, although less than Δ*mxiC*. Its own inducible secretion was reduced and that of all translocators slightly affected (Fig. 5*D*). These results suggest that a “straightened” MxiC favors the earliest step in the induction hierarchy, *i.e.* IpaD secretion, whereas a bent MxiC cannot prevent or favors, independently of its own secretion, the final one, *i.e.* effector release. In the wobbly mutant, three residues are altered that are initially identical to those changed in the straight one, but its phenotype is opposite, *i.e.* more like the bent one. This indicates that it is not the chemical nature of the amino acids in this surface patch that leads to these phenotypes but rather the stability of the secondary structure they form.

##### MxiC Is Not in Its Straight Conformation in Solution

The secretion patterns observed in the helix 9 mutants suggested movement in this area of MxiC is required for its function. We decided to examine the molecular conformation(s) of MxiC using double electron-electron resonance (DEER), also known as pulsed electron double resonance (PELDOR). This is an increasingly popular electron paramagnetic resonance (EPR) technique used to measure distances between paramagnetic spin labels on a nanometer scale (52).

Based on the MxiC structure (chain A of PDB entry 2VJ4 (21)) and the bent model (supplemental Fig. S8*A*), we chose residues Ala-247 and Ser-290 to introduce paramagnetic centers into the molecule as the distance between these residues was predicted to be in the measurable range for both forms, and the difference in distance between both forms was predicted as significant enough to be detectable (supplemental Fig. S9). To covalently couple the paramagnetic nitroxide label MTSL to these residues, Ala-247 and Ser-290 were mutated into cysteines. At the same time, the two endogenous cysteines Cys-184 and Cys-233 were mutated into alanine and serine, respectively, to finally generate a quadruple mutant *mxiC*(*C184A,C233S,A247C,S290C*) or *mxiC*(*Cys*). Alternatively, complementary mutant MxiCs, with cysteines for labeling inserted at two sets of different but equally suitable locations, could not be purified in sufficient amounts (data not shown).

The *mxiC*(*Cys*) behaved like the complemented strain in a CR secretion assay (supplemental Fig. S10). A His-tagged version of the cysteine mutant (His-MxiC(Cys)) was soluble when expressed in *E. coli* (supplemental Fig. S11*A*). We also combined the quadruple cysteine mutant with the previously generated bent (*mxiCV256P*) and straight (*mxiC*(*I251A,T253A,S254A,D255E*)) mutants. However, neither His-MxiC(bent,Cys) nor His-MxiC(straight,Cys) were solubly expressed in *E. coli* (data not shown). Thus, we could only analyze the “wild-type” His-MxiC(Cys).

**FIGURE 6.**
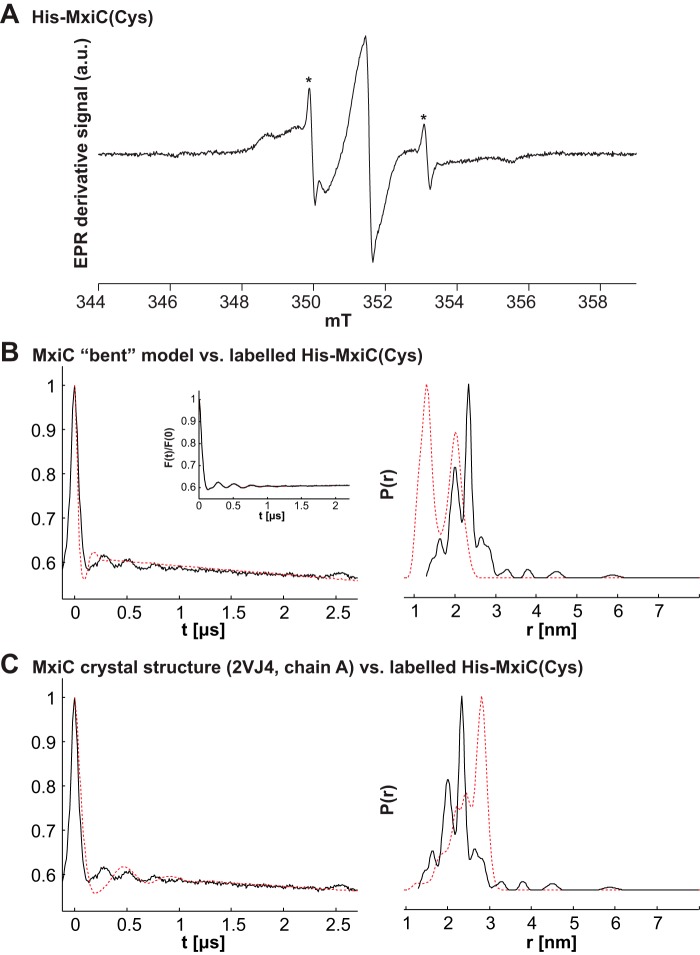
**His-MxiC(Cys) is nearly completely labeled with MTSL and the 247–290 interspin distance distribution is between that of the straight and bent forms.** His-MxiC(Cys) was modified with MTSL as described under “Experimental Procedures.” The labeling efficiency was assessed using room temperature continuous wave EPR. The resulting EPR derivative signal is displayed in arbitrary units (*a.u*.). *A*, labeling efficiency in His-MxiC(Cys) alone. The spin concentration is ∼ 66 μm, and ∼100% of His-MxiC(Cys) was modified by MTSL (the residual free label fraction is marked with an *asterisk*). *B* and *C, left column*, *V*(*t*)*/V*(0) is the primary DEER data; the *inset* in *B* shows the background-corrected *F*(*t*)/*F*(0) with the fit (*red dotted line*). In the *right column*, *P*(*r*) indicates the probability for the different distances extracted with model-free Tikhonov regularization using DeerAnalysis. The *red dotted lines* show the simulation by MMM. *B*, comparison of the bent MxiC model with the experimental data for His-MxiC(Cys). *C*, comparison of the MxiC crystal structure (PDB code 2VJ4, chain A) with the experimental data for His-MxiC(Cys). Comparisons with all other chains in all crystal structures are shown in supplemental Fig. S12. The chain presented in this panel was chosen because it was used as template to model the bent form of MxiC.

MTSL was covalently coupled to His-MxiC(Cys) at residues 247 and 290. The protein was concentrated and mixed with deuterated glycerol for EPR/DEER experiments (for details see under “Experimental Procedures”). The labeling efficiency was assessed using continuous wave room temperature EPR on a 35 μm sample (Fig. 6*A*). Using a previously obtained standard curve, the spin concentration was calculated to be 66 μm. The amount of residual unbound label was calculated to <1%; thus a spin labeling efficiency of close to 100% could be extracted. The spectrum also indicated the protein was folded; because of increased mobility in a disordered structure, an unfolded protein would result in a spectrum similar to that of the free label.

We subsequently performed a DEER experiment to determine the distance distribution of the paramagnetic labels on Cys-247 and Cys-290. We obtained a distance distribution between 1.5 and 3 nm with two prominent peaks at 2 and 2.4 nm (Fig. 6, *B* and *C*). Reliability of the two peaks comes from the high signal-to-noise *F*(*t*) trace. We simulated the expected distance distribution for the bent MxiC model (Fig. 6*C*) and the MxiC structure (PDB code 2VJ4, chain A; Fig. 6*B*) using MMM (35) and found the experimental data obtained for labeled His-MxiC(Cys) fit neither simulation well (Fig. 6). The major distance peak for the straight MxiC structure was around 3 nm, and under the bent MxiC model, the major expected distance is at ∼1 nm. As the distance simulations depend on the orientation of the side chains to calculate the probabilities of the label rotamers, we simulated the expected form factors and distances for all seven individual chains in the three MxiC crystal structures (PDB codes 2VIX, 2VJ4, and 2VJ5 (21)) (supplemental Fig. S12). Although the modeled form factors all differ in their overall shapes, they all show distances in the 1.5–3-nm range, in line with the experimental setup. However, the simulated peak at 3 nm was not as prominent in the experimental data. We thus conclude that the experimental data are not consistent with the structures nor with the bent model. When modeling the possible observable distances in the structure and the bent model using the “all rotamers” function in MMM (35), which neglects side-chain atoms, labeling of the chosen sites in both conformations can achieve a distribution close to the experimentally observed distribution (data not shown). Thus, the conformation(s) of purified MxiC in solution is consistent with both bent and straight forms.

##### IpaD Interacts with MxiC in Solution without Altering Its Conformation

**FIGURE 7.**
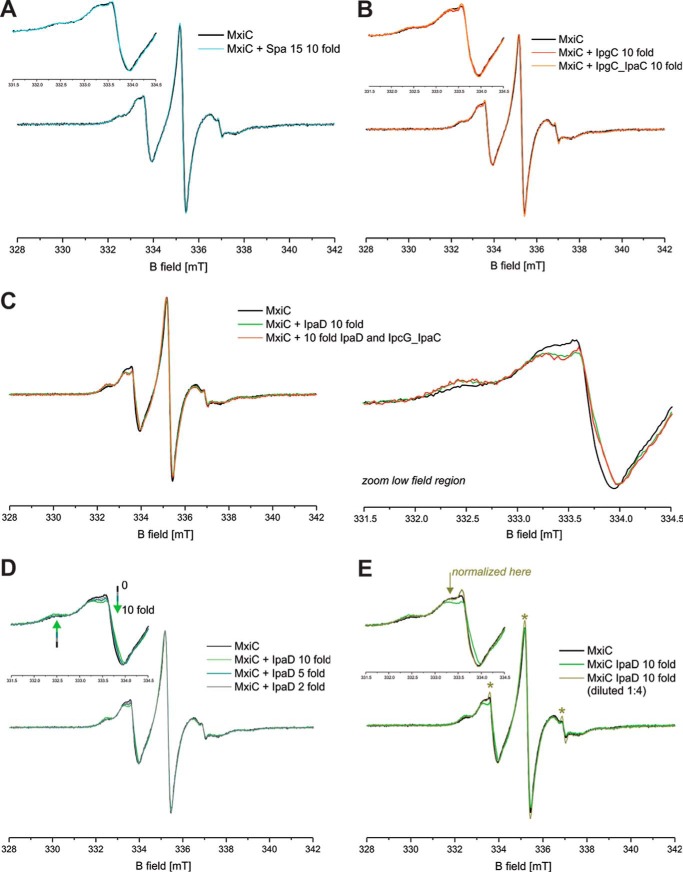
**Continuous wave EPR detects a weak but specific interaction of MxiC with IpaD.** *A*, cw EPR spectrum of spin-labeled MxiC in the absence (*black*) and in the presence of 10-fold excess of Spa15 (*cyan*). *B*, cw EPR spectrum of spin-labeled MxiC in the absence (*black*) and in the presence of 10-fold excess of IpgC (*red*) or IpgC-IpaC (*orange*). *C, left*, cw EPR spectrum of spin-labeled MxiC in the absence (*black*) and in the presence of 10-fold excess of IpaD (*green*) or IpaD-IpagC-IpaC (*red*). *Right*, the magnified low field region of the spectra (331.5–334.5 mT) highlights the detectable spectral differences. *D*, cw EPR spectrum of spin-labeled MxiC in the absence (*black*) and in the presence of *n*-fold excess of IpaD (indicated in *legend*). *E*, cw EPR spectrum of spin-labeled MxiC in the absence (*black*) and in the presence of 10-fold excess of IpaD (*green*) and after 1:4 dilution (*olive green*). The *inset* shows the magnified low field region of the spectra (331.5–334.5 mT), which highlights the fact that upon dilution the spectrum reverts to that of MxiC alone (except for a small fraction of free label, see *asterisks*), indicating that complex formation can be detected only at high protein concentrations due to the low affinity. The normalization of the spectral intensity of the diluted sample was done where indicated by the *arrow* to remove the effect of the free label. Except where otherwise stated, the spectra are normalized to the maximum amplitude of the central EPR line. The *insets* show the magnified low field region of the spectra (331.5–334.5 mT) to highlight possible spectral differences. The *asterisks* highlight the minor fraction of label, which is released during the incubation with some protein complexes and/or upon dilution.

As EPR only detects signals from paramagnetic centers, one can add potential binding partners to the sample without affecting the signal as long as they are diamagnetic. To determine whether other T3S-related proteins influence MxiC's conformation, we mixed labeled His-MxiC(Cys) with a 10-fold molar excess of the proposed interaction partner IpaD (15) or Spa15 (supplemental Fig. S11*B*), a protein that is not known to interact with MxiC (23). Spa15 addition did not change the continuous wave EPR spectrum (Fig. 7*A*), indicating Spa15 does not influence the rotational freedom of the labels in MxiC. Consistently, the DEER form factor and resulting distance distribution were also unchanged (data not shown). Thus, Spa15 does not affect MxiC's conformation *in vitro*.

**FIGURE 8.**
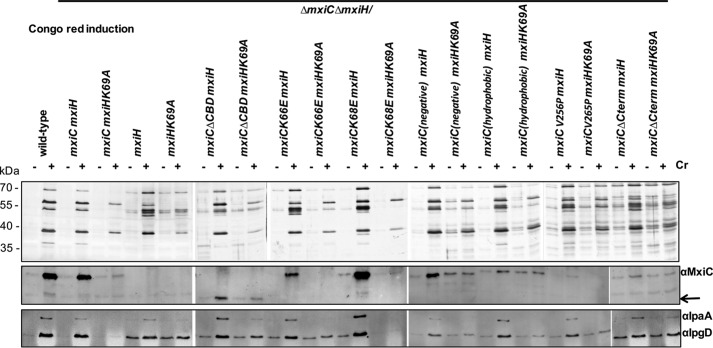
**Analysis of *mxiC* mutant phenotypes in the mxiHK69A background.** Protein secretion in response to the artificial inducer CR is shown. *Shigella* wild type, Δ*mxiC*Δ*mxiH* mutant, complemented strains (Δ*mxiC*Δ*mxiH*/*mxiCmxiH* or Δ*mxiC*Δ*mxiH*/*mxiCmxiHK69A*), and *mxiC* mutants (in the Δ*mxiC*Δ*mxiH* background expressing either *mxiH or mxiHK69A*) were grown with 25 μm IPTG where required. Mutants *mxiC*(*E201K,E276K,E293K*) and *mxiC*(*M226K,L242D*) are abbreviated *mxiC*(*negative*) and *mxiC*(*hydrophobic*), respectively. Samples were collected as described under “Experimental Procedures,” silver-stained (*top panel*), and Western-blotted with the indicated antibodies (*bottom panels*). *Arrow* indicates faster migration of *MxiC*Δ*CBD.*

In contrast, addition of a 10-fold molar excess of purified His-IpaD (supplemental Fig. S11*C*) changed the continuous wave (cw) spectrum obtained (Fig. 7*C*), indicating the conformational freedom of the spin-labeled side chains has been affected by IpaD-MxiC complex formation, either due to direct interaction with IpaD residues or to subsequent conformational changes in MxiC. To confirm IpaD was responsible for signal modification, we mixed MxiC with different concentrations of IpaD (Fig. 8*D*). We found that the larger the stoichiometric fold of IpaD *versus* MxiC, the bigger was the spectral change detected. This shows the signal modification is specific to IpaD addition and that, in the concentration range used, we can modify the ratio of the MxiC-IpaD complex *versus* MxiC alone in the equilibrium ensemble. We also diluted the 10:1 IpaD/MxiC mix 1:4 (Fig. 7*E*). This confirmed that in preventing the interaction between MxiC and IpaD by dilution, the MxiC alone signal is recovered, meaning the interaction is reversible.

Given the evidence that MxiC family proteins can interact directly with translocator chaperones (27, 36–38), we also purified the IpgC dimer and an IpaC-IpgC 1:1 heterocomplex (supplemental Fig. S11*D*) (39). Neither of these, added at 10-fold molar excesses, interacted with MxiC alone (Fig. 7*B*) or when added in the presence of a 10-fold molar excess of IpaD (Fig. 7*C*). Taken together, these data indicate that in solution only IpaD displays an affinity for MxiC. However, we found no evidence of a distance change between the two spin-labeled side chains by DEER upon addition of any protein partners (data not shown).

##### mxiC Mutants Unable to Prevent Effector Secretion Are Affected Differentially by Needle Mutant mxiHK69A, Which Is Unable to Release MxiC and Hence Effectors

To dissect at which point they are deregulated, we wanted to understand whether our *mxiC* mutants that leak effectors do so because MxiC's function(s) in blocking effector secretion is compromised or because they cannot retain MxiC intracellularly. We examined this by combining them with a mutation that prevents MxiC secretion, *mxiHK69A*.

An *mxiHK69A* mutant in the needle protein does not secrete effector proteins (16) nor MxiC (12). However, an *mxiHK69A* mutant lacking MxiC (Δ*mxiH*Δ*mxiC*/*mxiHK69A*) can secrete effectors (12). Thus, *mxiHK69A* does not physically block effector secretion, but it is unable to release MxiC's block of effector secretion.

When combining our *mxiC* mutants with *mxiHK69A*, MxiC mutants that cannot prevent secretion because the mutation(s) is affecting function should continue to leak effectors even if MxiC is retained inside bacteria. For instance, the hydrophobic core *mxiCF206S* mutant (Fig. 1) analyzed by Cherradi *et al.* (33) is not secreted in an *mxiHK69A* background but is still unable to block effector secretion. This indicates it has a severe defect in its inhibitory function that is independent of MxiC secretion. However, mutants where MxiC is functional but is secreted prematurely should prevent effector secretion when MxiC is forced to remain intracellular. To test this, we generated *mxiH* and *mxiHK69A* plasmids compatible with our *mxiC* mutant ones, which were then co-transformed into Δ*mxiC*Δ*mxiH* (see under “Experimental Procedures”). We focused solely on MxiC mutants displaying premature secretion of effectors and MxiC leakage. For completeness, we also included *mxiC*(*E201K,E276K,E293K*), which carries mutations in a negatively charged surface patch (Fig. 1, *top*), secretes translocators and MxiC prematurely, and is defective in IpaD secretion upon activation (15).

We were unable to detect leakage from Δ*mxiC*Δ*mxiH*/*mxiCmxiH* (data not shown), and its inducible secretion was also slightly reduced compared with wild type but of a similar level to that of Δ*mxiC*Δ*mxiH*/*mxiCmxiHK69A* (Fig. 8, *top panels*). Indeed, slightly fewer functionally mature T3SSs are assembled in Δ*mxiH*/*mxiHK69A* relative to wild type (16). However, as expected, Δ*mxiC*Δ*mxiH/mxiCmxiHK69A* could not leak or induce secretion of the effectors IpaA or IpgD nor of MxiC (Fig. 8, *bottom panels*), respectively. However, it secreted slightly reduced amounts of the translocators inducibly, as observed previously (16). In Δ*mxiC*Δ*mxiH/mxiHK69A*, IpaA and IpgD leakage was restored, and their secretion was also partially inducible (Fig. 8, *bottom panel*). We then examined how each *mxiC* mutant behaved in an *mxiHK69A* background. We focused on leakage in the absence of induction because activation of MxiC secretion is blocked in an *mxiHK69A* background but effector secretion might be allowed by a functionally defective MxiC.

Only *mxiCK68E* was unable to leak or induce effector secretion in the presence of *mxiHK69A* but not of *mxiH*. This indicates it is the only mutant where MxiC would be functional in blocking effector secretion, if it were not secreted prematurely. All other mutants leak effectors similarly whether *mxiH* or *mxiHK69A* is present, indicating that in these mutants MxiC's function(s) in blocking effector secretion are affected, either directly as in *mxiC*(*M226K,L242D*) or indirectly, via premature secretion of translocators as in *mxiC*(*E201K,E276K, E293K*).

## Discussion

### 

#### 

##### Classes of mxiC Mutants

Our mutants can be organized into classes according to their phenotypes (Table 1). However, residues mutated in the different classes do not obviously cluster in specific regions of the molecule (Fig. 1). Moreover, none seems affected only in a single step, *i.e.* regulation of translocator, MxiC, or effector secretion. This indicates the three regulatory functions of MxiC are interlinked. Therefore, the mutants were classified according to where their main initial defect lies, assuming the regulatory steps occur in this order.

**TABLE 1:**
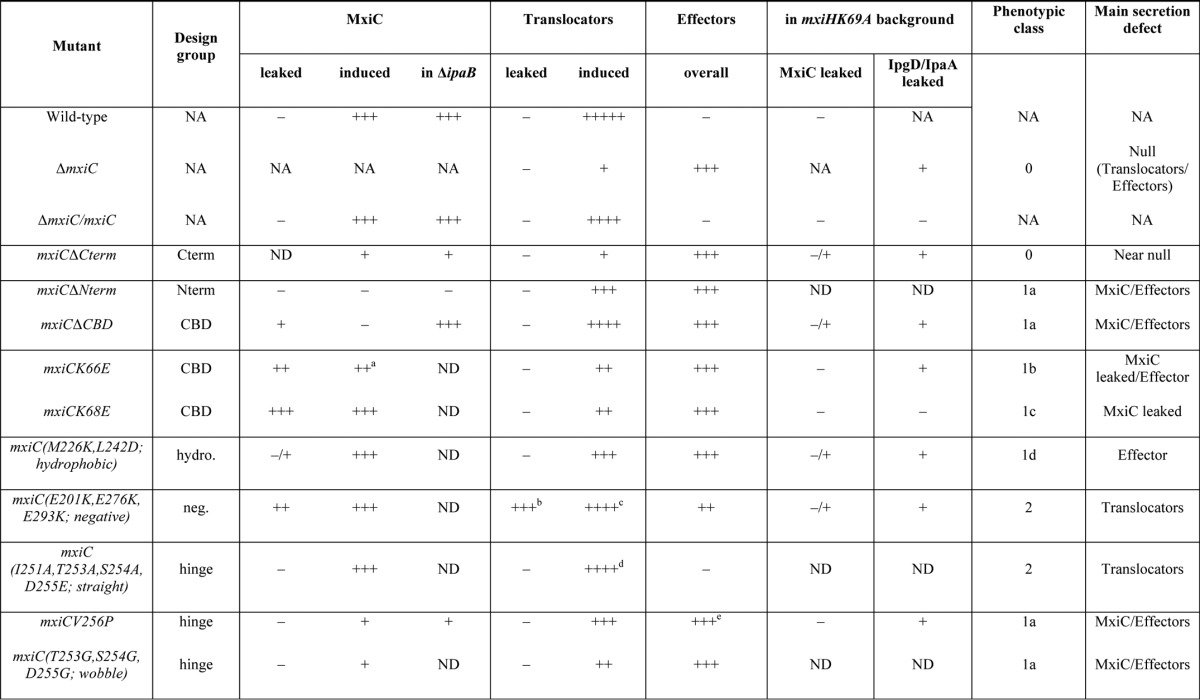
**Phenotypic overview of mutants** NA means not applicable, and ND means not determined.

*^a^* This indicares poorly detected in whole cell lysate.

*^b^* This indicares that IpaB/C increased.

*^c^* This indicares not IpaD.

*^d^* IpaC/B was reduced, IpaD slightly increased.

*^e^* IpgB1 was increased.

Only one mutant showed such severe loss-of-function as to resemble Δ*mxiC*, *mxiC*Δ*Cterm*, which we therefore placed in class 0. However, unlike *mxiC*Δ*Cterm*, *mxiC*Δ*Nterm* and *mxiC*Δ*CBD* were still largely able to induce translocator, but not MxiC, secretion, suggesting they are not full loss-of-function mutants (class 1a). Mutant *mxiCV256P* also could not secrete MxiC well inducibly or in a Δ*ipaB* background, suggesting it has some defect in MxiC secretion. This mutant leaks effectors, but as it can still stimulate secretion of translocators, it is also not a complete loss-of-function mutant (class 1a). Both classes of mutants readily by-passed the block on effector secretion imposed by *mxiHK69A*, although none secreted MxiC efficiently in this background, indicating they are unable to establish all or some of MxiC's regulatory functions.

*mxiCK66E* and *mxiCK68E* are phenotypically similar in that they secrete MxiC prematurely and hence show reduced translocator induction and increased effector leakage. However, MxiCK68E is secreted much more efficiently than MxiCK66E. Furthermore, among all mutants, only MxiCK68E does not leak effectors in an *mxiHK69A* background. This indicates that the mutations in MxiCK68E and MxiCK66E, despite their proximity and chemical similarity, lead to different defects. K68E (class 1c) primarily deregulates MxiC secretion, leading it and effectors to be secreted prematurely. In addition to some premature secretion, K66E (class 1b) seems to confer more fundamental defect(s) leading to effector leakage. Finally, the only defect seen in *mxiC*(*M226K,L242D*) is effector leakage (class 1d).

Class 2 mutants are primarily, if oppositely, affected in translocator release. MxiC(E201K,E276K,E293K) cannot induce IpaD secretion but leaks IpaB, IpaC, itself, and effectors (15), although MxiC (I251A,T253A,S254A,D255E) displays increased IpaD secretion and reduced IpaB and IpaC secretion.

##### Properties of MxiC and Roles of Its Termini

Using the mutant classes, we conclude that several physiological features of MxiC are regulated as follows: 1) its secretion, because several mutants up- or down-regulate it; 2) hierarchical secretion of translocators and effectors, and even of proteins within those two categories; and 3) the ability to switch from translocator to effector secretion. There are also uncharacterized fundamental properties of MxiC as indicated by mutations that most likely do not affect its overall structure, *i.e. mxiC*Δ*Cterm* and *mxiC*Δ*Nterm*, but still lead to full or partial null phenotypes, respectively.

MxiC's secretion signal and putative CBD are dispensable for stimulation of translocator secretion, which indicate domains 1–3 alone mediate this function. Multiple residues in the C-terminal helix of MxiC are highly conserved. Schubot *et al.* (20) suggested that the C-terminal helix of *Yersinia* TyeA (a MxiC domain 3 homolog that binds the C terminus of YopN, a MxiC domains 1–2 homolog) might localize the YopN-TyeA complex to the secretion apparatus. Ferracci *et al.* (19) found TyeA to be essential for YopN to block secretion. In addition, several mutants in the hydrophobic core of domain 2 (Fig. 1) of MxiC are non-functional or at least unable to block secretion (33). In contrast, equivalent mutations in *Yersinia yopN* lead to a general block of secretion (19, 33). However, the *yopN* mutants analyzed by Ferracci *et al.* (19) were overexpressed in comparison with wild-type levels. In our hands, overexpression of the non-functional hydrophobic core of domain 2 *mxiCD225V* mutant (Fig. 1) also led to a general block of secretion (data not shown). This suggests MxiC blocks an acceptor site on the secretion apparatus, and too much non-removable protein prevents release of this repressor, consequently blocking secretion. In support of this, Lee *et al.* (40) showed Pcr1, the *Pseudomonas* T3SS homologs of *Yersinia* TyeA, interacts with the T3SS inner membrane protein PcrD, known as MxiA in *Shigella*. Furthermore, the extreme C terminus of MxiC is required for interaction with the cytoplasmic region of MxiA (41), explaining the null phenotype of *mxiC*Δ*Cterm*. As mutants in the N terminus and CBD are unable to secrete MxiC inducibly and an N-terminal His tag on MxiC led to a Δ*mxiC* phenotype (data not shown), these regions may also be involved in binding it to and releasing it from that site.

##### MxiC Differentially Affects Secretion of IpaD and Hydrophobic Translocators

Translocator secretion was largely unaffected in class 1a and 1d mutants, although all had clear defects in blocking effector secretion. In contrast, mutants from class 2 modulated translocator secretion, but effector secretion was affected indirectly (*mxiC*(*E201K,E276K,E293K*)) or not at all (*mxiC*(*I251A,T253A,S254A, D255E*)). Thus, the action of MxiC on translocators and effectors can be uncoupled. MxiC(E201K,E276K,E293K) acts as if it had already received the activation signal (15). Thus, MxiC's action on translocators can also be uncoupled from the needle-transmitted activation signal. In addition, MxiC secretion is not required for activating translocator secretion, as an *mxiHK69A* mutant, which does not secrete MxiC, and *mxiC*Δ*Nterm* secrete normal levels of translocators (12, 16).

Even hydrophilic *versus* hydrophobic translocator secretion can be uncoupled because *mxiC*(*E201K,E276K,E293K*) leaks IpaB and IpaC and is unable to induce IpaD secretion (Roehrich *et al.* (15)). Interestingly, the straight mutant *mxiC*(*I251A,T253A,S254A,D255E*) instead displays reduced induction of IpaB and IpaC, although IpaD secretion is slightly increased. These opposite effects correlate with the mutations being on opposite faces of MxiC (Fig. 1, *top*). These faces are also the ones that may undergo a conformational change from flat to bent (21). In fact, this is the conformational change we tried to prevent in *mxiC*(*I251A,T253A,S254A,D255E*). Furthermore, the predicted bent mutant (*mxiCV256P*) and the predicted straight mutant show opposite phenotypes; although the former leaks effectors and is only weakly affected in translocator secretion, the latter shows differential induction of translocators. Thus, reduced secretion of the repressor IpaD could affect secretion of the hydrophobic translocators. Alternatively or additionally, MxiCV256P was only poorly secretable and could be affected in more fundamental functions. Although we cannot exclude that these mutations mainly affect MxiC interactions by altering its surface, the type of mutations, the fact that the mutations are directly adjacent, and their opposite phenotypes suggest that we have modified MxiC's ability to undergo conformational changes.

##### Conformational Change in MxiC?

The negatively charged patch mutated in *mxiC*(*E201K,E276K,E293K*) lies on the face of the molecule that is flat in MxiC but concave in YopN/TyeA and the *E. coli* homolog SepL (26). As the straight mutant favored IpaD secretion, a flat negatively charged patch might be required for this. Lower levels of IpaB and IpaC secretion could then be due to the restricted conformation or to the mutations themselves. In other words, IpaB and IpaC secretion could require either a bent conformation or the area in helix 9 that was mutated in *mxiC*(*I251A,T253A,S254A,D255E*), or both. The latter is supported by the work of Archuleta and Spiller (27), which indicates that a flattened gatekeeper structure has a conserved binding site for translocator chaperones, such as IpgC in *Shigella*, at this location. In the *Salmonella* SPI-1 system, the MxiC homolog InvE interacts with complexes of hydrophobic translocators and their chaperone (36). An interaction between MxiC or its homolog and the class 2 chaperone IpgC or its homolog has also been shown in *Shigella* and *Chlamydia* (33, 38). Finally, we identified substoichiometric amounts of IpgC and all translocators in our interaction partner screen (32).

##### MxiC Directly Interacts with IpaD

Unfortunately, we were to unable to test for any conformational change in MxiC by EPR. However, we did detect an interaction between MxiC and IpaD via this method. The spectral changes observed in the continuous wave EPR experiments where MxiC(Cys) and IpaD were mixed have two possible explanations as follows: (i) the increased molecular weight of the complex(es), which is reflected in slower overall rotational correlation times; (ii) a direct interaction between the spin-labeled probes and residues in IpaD. Although the reason of the spectral effects observed cannot be clarified, it corroborates the notion that complex formation between MxiC and IpaD occurs. From Fig. 7*B*, assuming the 1:10 MxiC/IpaD concentration corresponds to the maximum amount of complex formation and therefore that a 1:5 molar ratio IpaD gives half-maximum complex concentration, we estimate the dissociation constant of the MxiC-IpaD complex is ∼730 μm, *i.e.* these purified proteins have very low affinity. This suggests we have not fully reconstituted interactions between these proteins *in vitro*. We may be lacking their “scaffold.” Does the MxiA_C_ oligomer, aided by other T3SS export apparatus components, perhaps stabilize one of MxiC's conformations?

**FIGURE 9.**
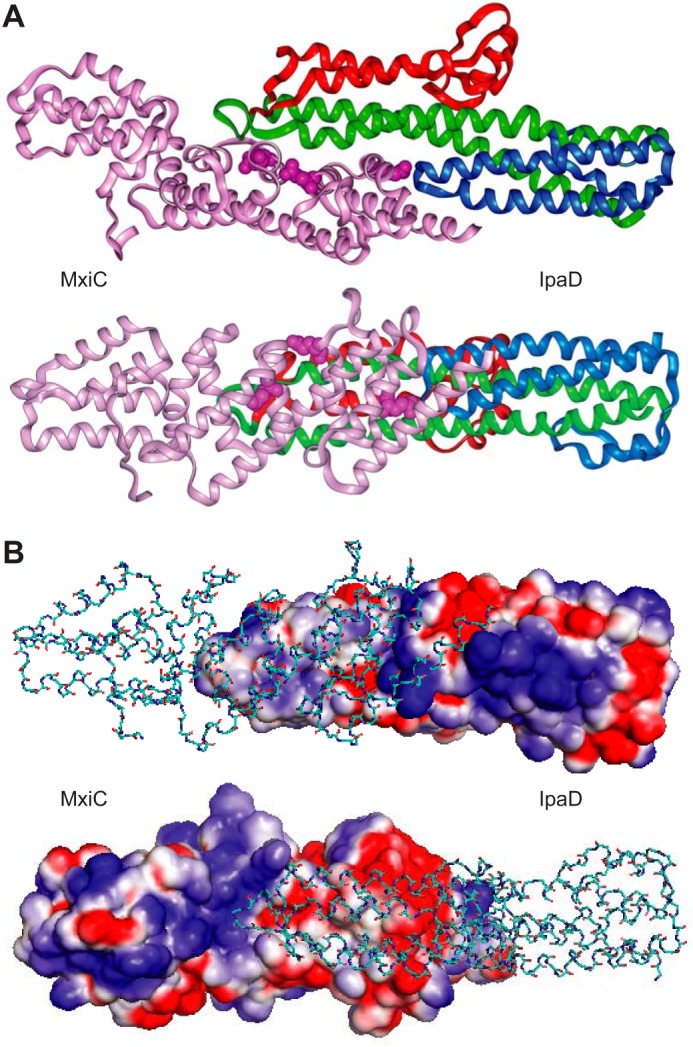
**Modeling of MxiC-IpaD interaction.** *A*, docking of gray MxiC (PDB entry 2VJ4 chain A (residues 64–349) (21), *pink*) and colored IpaD (PDB entry 2J0O chain A (42)). The N-terminal domain of IpaD is colored in *blue* (residues 40–130); the C-terminal globular domain is colored in *red* (residues 177–271); and the coiled-coil domain is colored in *green* (residues 131–176 and 272–321). Residues Glu-201, Glu-276, and Glu-293 involved in the negatively charged patch of MxiC are highlighted as space-fill model. The *bottom panel* is rotated 90° around the *x* axis compared with the *top panel* illustrating the elongated shape of the dimer. *B*, interface of the MxiC IpaD dimer shows complementary charges. The *top panel* shows the electrostatic potential surface of IpaD and the backbone of MxiC, and the *bottom panel* shows the electrostatic potential surface of MxiC and the backbone of IpaD. The panels are rotated 180° with respect to each other. Negative charges are shown in *red* and positive charges are depicted in *blue*. The docking was performed manually using the location of *ipaD* and *mxiC* mutants. The model was generated in Insight II 2005 and optimized using Discover 2.98. Electrostatic potential surfaces were calculated using Delphi (all Accelrys Inc.).

Based on the crystal structures of IpaD (42) and MxiC (21) and the mutations genetically affecting the interaction between these proteins (*ipaDL99P* and *mxiC*(*E201K,E276K,E293K*) (15)), we built a model of the MxiC-IpaD interaction; the MxiC and IpaD structures pack readily against each other (Fig. 9*A*), with the negatively charged patch of MxiC (Glu-201, Glu-276, and Glu-293) at their interface, yielding an overall elongated form. The interacting surface is relatively flat and contains several charged residues on the edges and stacked tyrosine residues in the center giving an interface formed by two charge-complementary surfaces (Fig. 9*B*). This model is consistent with the data of Lee *et al.* (40), who found that *Pseudomonas* PcrG, a homolog of the N terminus of IpaD, interacts with Pcr1, a MxiC domain 3 homolog. Taken together with the work of Archuleta and Spiller (27), our model suggests why MxiC's N terminus and CBD are dispensable for stimulating translocator secretion.

##### Intramolecular and External Signals for MxiC Secretion

By analyzing the *mxiC*Δ*Nterm* mutation in the constitutive secretor background Δ*ipaB*, we confirmed that this mutant is unsecretable. Similar to the N-terminal secretion signal, the putative chaperone-binding domain is required for efficient MxiC secretion. However, this domain did not affect secretability. Thus, MxiC secretion is merely deregulated in *mxiC*Δ*CBD*. Interestingly, two different mutations in the CBD of MxiC led to increased MxiC leakage, *mxiCK68E* and *mxiCK66E*. Taken together, these data suggest that the putative chaperone-binding domain positively regulates MxiC secretion.

The chaperone-binding domain is irrelevant for MxiC secretion in the Δ*ipaB* background. This is reminiscent of the lack of requirement of MxiC for translocator secretion in the same background. Similar to the repression of translocator secretion (12), an intracellular repressor mechanism for MxiC secretion might be put into place once the tip is fully assembled; the CBD would be involved in counteracting this mechanism, and mutants *mxiCK68E* and *mxiCK66E* would then either be better at counteracting the mechanism repressing MxiC secretion or less sensitive to it.

The “external” signal for MxiC secretion has not yet been identified. However, this signal is likely transmitted through the needle as an *mxiHK69A* mutant is unable to release MxiC even when secretion is artificially activated by additional deletion of *ipaB* (12). The question of how *mxiHK69A* blocks MxiC secretion remains; the *mxiHK69A* mutant is either unable to transmit an external activation signal for MxiC secretion generated after the translocators have been secreted or this mutation alters the affinity of the secretion apparatus for MxiC (12, 32).

Mutants *mxiCK68E* and *mxiCK66E* leaked the respective mutant protein and hence probably overcame an intracellular repression mechanism for MxiC secretion. Similarly, *mxiC*(*E201K,E276K,E293K*) has gained the ability to activate its own secretion (15). However, none of these mutants is inducibly secreted in the *mxiHK69A* background. Indeed, we did not identify any mutation allowing induction of MxiC secretion in the *mxiHK69A* background. Thus, in addition to blocked transmission of Signal 2, the secretion apparatus in the *mxiHK69A* mutant was unable to release/recognize MxiC as a secretion substrate.

##### MxiC Secretion per se Is Not Required to Derepress Effector Secretion

In the wild type, the needle transmits the activation signal to MxiC, whose subsequent secretion leads to derepression of effector secretion. Class 1a mutants, which only secrete MxiC poorly but leak effectors, show that effector secretion and MxiC secretion can also be uncoupled. In other words, MxiC secretion *per se* is not required for allowing effector secretion. Not all MxiC homologs are secreted (28). However, the gatekeepers must be removed from their initial place of action; in the *Salmonella* SPI-2 system where the MxiC homolog SsaL is not secreted, the protein dissociates from the membrane and is degraded once secretion is activated (43).

##### Are There Further Regulatory Function(s) for MxiC?

Mutant *mxiCV256P* leaked effectors, including very high levels of IpgB1. IpgB1 is an effector protein chaperoned by Spa15 (24, 44). The specific effect of *mxiCV256P* on IpgB1 suggests that MxiC somehow directly affects secretion of at least some effectors. Similarly, *E. coli* SepL was shown to specifically act on the effector Tir. This protein-protein interaction was proposed to be critical for regulating secretion of the other effector proteins (28). Although this does not exclude a possible effect of MxiC on effector secretion in a more general way, *e.g.* by modulating the affinity or physically blocking an acceptor site for effectors in the T3SS, it suggests that secretion of specific effectors involves an additional regulatory layer.

##### Steps in MxiC Function

After T3SS assembly, but before secretion activation, multiple mechanisms are in place that prevent premature secretion of the different protein types. Translocator secretion is repressed by IpaD, probably via interaction with MxiC bound to MxiA. An uncharacterized mechanism prevents MxiC secretion, although this protein itself prevents effector secretion. When the needle tip comes in contact with a host cell, an activation signal (“Signal 1”) is transmitted to the cytoplasm, most likely through the needle.

The mechanism preventing premature translocator secretion is counteracted by MxiC. Secretion of IpaD requires the negatively charged patch and probably a flat conformation of the molecule. Removal of IpaD may initiate a conformational change in MxiC, which bends to release the IpgC chaperone and its bound translocators allowing these to become secreted in turn. IpaB and secreted IpaC form a pore, connected to IpaD at the needle tip and also inserted in the host cell membrane, which transmits “Signal 2” to the cytoplasm. Reception of this signal may allow finalization of a conformational change in MxiC that releases it for secretion. Then, early effectors are secreted. Moreover, as both negative regulators are now removed, the secretion rate is enhanced (40), possibly through alteration of its mode (41).

By using structure- and sequence-based mutagenesis of *mxiC*, we dissected at which stages of the regulatory cascade MxiC acts, including which of its functional steps can be uncoupled. This also allowed us to determine how the different steps are connected. Finally, we provide the first evidence that a directionally defined conformational change in MxiC is involved in controlling the secretion hierarchy. The conservation of the components involved indicates the importance of this regulatory pathway to T3SS-carrying bacterial pathogens of animals.

## Experimental Procedures

### Bacterial Strains, Plasmids, and Primers

The supplemental Tables S1 and S2 list the strains and plasmids used. *S. flexneri* strains were grown in Trypticase Soy Broth (BD Biosciences) at 37 °C with appropriate antibiotics the following final concentrations: ampicillin 100 μg ml^−1^, kanamycin 50 μg ml^−1^, tetracycline 5 μg ml^−1^, and chloramphenicol 10 μg ml^−1^. IPTG was used at the final concentrations indicated in the figure legends. The supplemental Table S3 lists the primers used.

### Construction of Plasmids

Plasmids were generated as described below and verified by sequencing.

pIMA221 (pWSK29*mxiC) was generated by amplifying *mxiC* from the *Shigella* virulence plasmid pWR100 using primers mxiC_SacI and mxiC_BamHI. The purified PCR product was digested with SacI and BamHI and cloned into pWSK29* (modified pWSK29 (45) lacking the T7 promoter, a gift from Andrew Davidson (University of Bristol) digested with the same enzymes.

#### 

##### Terminal Deletions and Mutations

To express the terminal *mxiC* deletions, *mxiC* was amplified from pIMA221 using primers mxiC_SacI_del30 and mxiC_BamHI for *mxiC*Δ*Nterm* and primers mxiC_SacI and mxiC_341 for *mxiC*(*1–341*). The resulting PCR products were digested with SacI and BamHI. Fragments *mxiC*Δ*Nterm* and *mxiC*(*1–341*) were cloned into pACT3 digested with SacI/BamHI yielding pDR60 and pDR73, respectively.

##### Chaperone-binding Domain Mutants

pDR80 contains *mxiC*Δ*CBD*, an internal deletion mutant that was created by two-step PCR and lacks residues 32–72. The *mxiC* gene was amplified from pIMA208 using primer pairs mxiC_SacI/mxiC_D32–72_R and mxiC_D32–72_F/mxiC_BamHI. The obtained PCR fragments were used as template for the second PCR step using primers mxiC_SacI and mxiC_BamHI. The PCR product was purified, digested with SacI/BamHI, and ligated into pACT3 digested with the same enzymes.

Mutant *mxiCK66E* was generated by two-step PCR. First, *mxiC* was amplified from pIMA208 using primers mxiC_SacI and mxiC_K66E_R and/or mxiC_K66E_F and mxiC_BamHI, respectively. These fragments were combined and reamplified using mxiC_SacI and mxiC_BamHI. The PCR products were purified, digested with SacI and BamHI, and cloned into pACT3 digested with the same enzymes yielding pDR96. pDR100 contains *mxiCK68E* cloned via SacI/BamHI, equivalent to amplification of mutant *mxiC* using primers mxiC_SacI and mxiC_BamHI. This mutant was obtained by chance and retained as the mutation was in a relevant area of the molecule.

##### Hydrophobic Patch Mutant

Double mutant *mxiC*(*M226K,L242D*) was generated by two-step PCR. pIMA212 was used as template for reactions with primer pairs mxiC_SacI/mxiC_M226K_R and mxiC_L242D_F/mxiC_BamHI. The obtained fragments were combined and reamplified using primers mxiC_SacI and mxiC_BamHI. The product was purified, digested with SacI and BamHI, and cloned into pACT3 digested with the same enzymes, yielding pDR72.

##### Hinge Region Mutants

Mutants in helix 9 were generated by two-step PCR; first, mxiC was amplified from pIMA208 using primer pairs mxiC_SacI/mxiC_V256P_R, mxiC_V256P_F/mxiC_BamHI, mxiC_SacI/mxiC_wobble_R, mxiC_wobble_F/mxiC_BamHI, mxiC_SacI/mxiC_straight_R2, and mxiC_straight_F2/mxiC_BamHI. The obtained PCR fragments were used as template for the second PCR using primers mxiC_SacI and mxiC_BamHI. The products were purified and digested with SacI/BamHI before ligation into pACT3 digested with the same enzymes. The resulting plasmids were named pDR91 (pACT3mxiCV256P), pDR92(pACT3mxiC(T253G,S254G,D255G)), and pDR93(pACT3mxiC(I251A,T253A,S254A,D255E)), respectively. The background quadruple cysteine mutant gene *mxiC*(*Cys*) (C184A (TGT to GCA), C233S (TGT to TCT), A247C (GCA to TGT), and S290C (AGT to TGT)) required for EPR experiments was synthesized by MWG Eurofins and supplied as pEXA-mxiC_EPR2. For pDR104 (pET28b*mxiC*(*Cys*)), *mxiC*(*Cys*) was amplified from the pEX-A-mxiC_EPR2 using primers mxiC_NdeI_F/mxiC_EcoRI_R. The PCR product was purified and digested with NdeI/EcoRI before cloning into pET28b (Novagen).

### Chaperone Mutants

Deletions of the chaperone genes were performed using the method of Datsenko and Wanner (46). A kanamycin cassette was amplified from pKD4 using primers ipgA_KO_kanF/ipgA_KO_kanR, ipgE_KO_kanF/ipgE_KO_kanR, and spa15_KO_kanF/spa15_KO_kanR, respectively. The primers contained ∼50 bp upstream and downstream of the respective chaperone gene to allow for recombination by the λ Red recombinase. These fragments were introduced into *Shigella* wild type yielding Δ*ipgA*, Δ*ipgE*, and Δ*spa15*, respectively. For Δ*ipgE* Δ*spa15* (abbreviation of Δ*ipgE*::FRT Δ*spa15*::kan) and Δ*ipgE* Δ*ipgA* (abbreviation of Δ*ipgE*::FRT Δ*ipgA*::kan), the kanamycin cassette was removed in Δ*ipgE* by FLP-mediated recombination using the introduced FRT sites yielding Δ*ipgE*::FRT. The same fragments used for the deletion of *spa15* and *ipgA* in the wild-type background were now used for λ Red recombination in Δ*ipgE*::FRT. This step was of low efficiency as the recombination preferentially occurred at the FRT scar at the Δ*ipgE* site and not up- and downstream of *spa15* and *ipgA*, respectively. Colonies were prescreened by colony PCR using primers annealing ∼200–300 bp upstream and downstream of the original chaperone gene, seeking to obtain a PCR fragment with a size compatible with the insertion of the kanamycin cassette *versus* the chaperone gene. Δ*ipgE* Δ*ipgA* Δ*spa15* (abbreviation of Δ*ipgE*::FRT Δ*ipgA*::FRT Δ*spa15*::kan) was generated by removing the kanamycin cassette in Δ*ipgE* Δ*ipgA* by FLP recombination yielding Δ*ipgE*::FRT Δ*ipgA*::FRT and subsequent λ Red recombination using the ipgA::kan PCR fragment. Again, this step was of low efficiency as now two FRT sites were available on the virulence plasmid and colonies were prescreened by colony PCR as described for the double knockouts. All insertions and cassette removals were verified by sequencing.

### Combination of mxiC and mxiH Mutants

Some of the *mxiC* plasmids detailed above were then combined with plasmids carrying *mxiHK69A* or wild-type *mxiH* as control in Δ*mxiC*Δ*mxiH* (12). *mxiH* and *mxiHK69A* were amplified from corresponding templates (16), using primers mxiH_NdeI_For and mxiH_PstI_Rev, and cloned into a previously described pUC18 vector, modified to carry a constitutive *lac* operator (14). This was done so when both pACT3 containing *mxiC* and pUC18 containing *mxiH* were transformed into Δ*mxiC*Δ*mxiH* bacteria, *mxiH* expression would not be inhibited by LacI, encoded on pACT3, binding to the *lac* operator of pUC18. The corresponding bacteria had normal expression of *mxiH* and *mxiC* in the presence of 25 μm IPTG except Δ*mxiC*Δ*mxiH/mxiC*Δ*Nterm mxiH* wild-type or K69A when 100 μm IPTG was used to ensure sufficient expression of this *mxiC* mutant.

### Type III Secretion Functional Assays

#### 

##### Analysis of Protein Expression Levels

Whole cultures of *S. flexneri* in late exponential phase (*A*_600_ ∼1) were mixed with 4× Laemmli sample buffer (“whole culture lysates”). Samples from equivalent cell numbers (∼3 × 10^6^ cfu) were separated by 10% SDS-PAGE and immunoblotted.

##### Analysis of Leakage

*S. flexneri* strains grown to *A*_600_ ∼1 were collected by centrifugation at 15,900 × *g* for 10 min at 4 °C. The supernatants were mixed with 4× Laemmli sample buffer, normalized for equivalent cell numbers, separated on 10% SDS-PAGE, and visualized by silver staining or immunoblotting. On silver-stained SDS-PAGE, labels indicate the position of proteins as determined by mass spectrometry or from deletion strains.

##### Analysis of Inducible Protein Secretion

CR, a small amphipathic dye molecule, is an artificial inducer of T3S. Its addition to a *Shigella* culture leads to a burst of Ipa protein secretion called “induction” (47, 48). *S. flexneri* at *A*_600_ ∼1 was collected by centrifugation at 4500 × *g* and resuspended in PBS to an *A*_600_ of 5, *i.e.* ∼1.5 × 10^9^ cfu ml^−1^. For each strain, two reaction tubes were prepared with 500 μl of bacterial suspension. To one of the tubes, CR (Serva) was added to a final concentration of 200 μg ml^−1^. After incubation at 37 °C for 15 min, samples were centrifuged at 15,900 × *g* for 10 min at 4 °C. 20 μl of the supernatants denatured in Laemmli sample buffer were subjected to 10% SDS-PAGE and visualized by silver staining or immunoblotting.

### Western Blotting

Proteins were transferred onto Immobilon FL (Millipore) membrane using a semi-dry method. Primary antibodies used were as follows: anti-IpaA mouse monoclonal, gift from Kirsten Niebuhr (3); anti-IpaB mouse monoclonal, named H16, gift from Armelle Phalipon (49); anti-IpaC mouse monoclonal, mixture of J22 and K24, gift from Armelle Phalipon (50); anti-IpaD rabbit polyclonal, gift from Claude Parsot (51) or as described in Cheung *et al.* (14); anti-IpgD mouse monoclonal, gift from Kirsten Niebuhr (3); and anti-MxiC rabbit polyclonal, raised against a fragment of MxiC containing residues 74–355 and an N-terminal His tag (12). Near-infrared fluorescent secondary antibodies (rabbit IgG raised in goat and coupled to Alexa680, Invitrogen; mouse IgG raised in goat and coupled to DyLight800, Pierce) were visualized and quantified on a LI-COR Odyssey imaging system.

### Calculation of the Secreted Percentage of a Protein

Wild-type *Shigella* were grown to *A*_600_ ∼1 and resuspended in PBS to an *A*_600_ of 15. The suspension was brought to 37 °C in a water bath before CR was added at a final concentration of 200 μg ml^−1^, and the cultures were incubated for 8 min. For secreted proteins, samples were centrifuged at 15,000 × *g* for 10 min at 4 °C, and supernatants were denatured in Laemmli sample buffer. For whole cultures, the bacterial suspension was directly denatured in sample buffer. To calculate the secreted percentage of each protein, we compared protein amounts in supernatants and whole cultures by Western blotting. Undiluted and 1:4 diluted supernatant was compared with a dilution series of the whole cultures. Near-infrared fluorescent secondary antibodies were quantified on a LI-COR Odyssey imaging system. A linear fit of the dilution series allowed us to determine the concentration of protein in the supernatant in comparison with the whole culture.

### EPR Spectroscopy

#### 

##### Modeling of the Bent MxiC Structure

To create a model of the putative bent form of MxiC, its straight crystal structure (PDB code 2VJ4 (21)) and the structure of YopN/TyeA (PDB code 1XL3, Schubot *et al.* (20)) were used. In a first step, MxiC was superimposed on YopN and TyeA independently. The majority of the first two X-bundle domains of MxiC (residues 64–253) were matched to YopN, and the C-terminal domain of MxiC (residues 254–355) was matched to TyeA. In the second step, the respective MxiC domains were taken, and the link between them (residues 250–260) was rebuilt manually, guided by the conformation of the equivalent region in YopN. This model was soaked in a 1-nm layer of water molecules and relaxed with 5000 steps of energy minimization under the Cvff force field. Insight II 2005 was used for the modeling and Discover 2.98 (both Accelrys Inc.) for the energy calculations.

##### Modeling of Interspin Distances

The spin label MTSL is relatively long and flexible: the linker to the protein backbone contains five dihedral angles. Thus, depending on the rotameric state of each spin label in a protein, the interspin distance can vary significantly. When bound to a protein, the spin label's rotamers have different energies due to their interactions with the neighboring side-chains of the protein. The most favorable rotamers and thus the most likely distance distribution were calculated using MMM version 2013, a Matlab package (35). We used eight different MxiC structures to calculate the interspin distance distribution; the bent MxiC model and all seven MxiC crystal structures from Deane *et al.* (21) (PDB codes 2VIX, 2VJ4, and 2VJ5) were extracted so that only a single polypeptide chain was present in each PDB file. This was necessary as the labeled residues (247 and 290) are in close proximity to the other polypeptide chains in the original PDB files. The neighboring chains thus also influence the rotamer modeling; however, MxiC is most likely monomeric in solution (21), and therefore these additional interactions are not meaningful and were excluded.

##### Protein Purification

Each protein was purified first by nickel affinity chromatography and then by size-exclusion chromatography. The protocols for these purifications were based on articles herewith: Spa15 (53); IpgC/IpaC (39); and MxiC(C184A/C233S/S243C/S290C) (21). For IpaD, a C322S mutant was used to avoid the requirement for DTT addition during the purification (54). For this, His_6_-IpaD(15–332)_C322S_ was amplified from pUC18 ipaD_C322S_ (14), using primers ipaD15_NdeI_For and ipaD_BamHI_Rev (supplemental Table S3), and cloned into pET15b (Novagen) via NdeI/BamHI. The changes to the protocol for each protein are detailed in supplemental Table S4.

All protein concentrations, measured with a Nanodrop Lite spectrophotometer (Thermo Scientific), were adjusted using molecular weight, extinction coefficient (as obtained from the ExPASy server) of the protein, and consideration of the path length. Prior to each protein purification, the appropriate bacterial strain was streaked out on an Lysogeny Broth (LB) agar plate containing the appropriate antibiotics and grown overnight at 37 °C. The next day small LB cultures were made overnight. In the morning, cultures were made with overnight cultures (supplemental Table S4). Bacteria were grown to an *A*_600_ of ∼0.6 before cooling to 20 °C and induction with a final concentration of 1 mm IPTG. Cultures were then left shaking at 20 °C overnight.

Cells were harvested by centrifugation (5000 × *g* for 15 min), washed in 10 ml of PBS (3500 × *g* for 5 min), and resuspended with ∼30 ml of Binding buffer with protease inhibitors (Complete EDTA-free, Roche Applied Science). Bacteria were lysed by sonication (Sonics Vibra Cell^TM^) at amplitude 60%, pulse 1-s on and 1-s off, time of 30 s, and variable cycle numbers (supplemental Table S4).

Lysates were clarified by centrifugation for 30 min at 20,000 × *g* at 4 °C. Supernatants were filtered through a 0.45-μm and then 0.22-μm syringe filter (Sartorius) and applied to a 5-ml HisTrap FF column (GE Healthcare) equilibrated in binding buffer (supplemental Table S4) using a peristaltic pump (GE Healthcare). The entire supernatant was passed over the column three times at 2.5–5 ml/min at 4 °C. The column was then connected to an ÄKTA (GE Healthcare) and washed with 10–15 column volumes of Binding buffer, followed by elution buffer (supplemental Table S4). Elution of bound proteins was carried out in the presence of protease inhibitors, with an imidazole gradient from 20 mm to 1 m (Elution buffer; supplemental Table S4) to 3 ml/min for 25 min. 5-ml fractions were collected. Peak fractions potentially containing proteins of interest had their concentration determined at *A*_280_ and were examined using Coomassie-stained SDS-polyacrylamide gels. The appropriate fractions were pooled and concentrated using Amicon Ultra spin concentrator (molecular cutoff, Millipore; supplemental Table S4).

These concentrated fractions were run on a Superdex75 10/30 size exclusion chromatography column (GE Healthcare) equilibrated in 20 mm Tris-HCl, pH 7.5, 100 mm NaCl, and 100–250-μl samples were applied, and the column was run at 0.5 ml/min collecting 1-ml fractions. Location and purity of proteins of interest were verified by Coomassie-stained SDS-PAGE. After the final gel filtration step, all samples were concentrated as above (supplemental Table S4) and flash-frozen in aliquots in liquid nitrogen and stored at −80 °C until use.

##### MxiC Labeling

1 mm freshly made DTT (Sigma) was added to MxiC(C184A/C233S/A247C/S290C) to reduce the SH of the cysteines before concentration and application on the gel filtration column. The fractions containing His-MxiC(C184A/C233S/A247C/S290C) (∼2.12 mg/ml) were pooled, and the spin label MTSL (Toronto Research Chemicals, Toronto, Canada) was added at 2-fold molar excess (∼121 μm). This means one molecule of MTSL was added per cysteine in the sample. After incubation for overnight at 4 °C in the dark, the sample was concentrated to ∼1.842 mm (∼70 mg/ml) as determined by the *A*_280_ using Amicon Ultra-4 spin concentrators (10-kDa molecular mass cutoff, Millipore).

##### Determination of the Spin Concentration and Labeling Efficiency Using Continuous Wave EPR

To determine the labeling efficiency of His-MxiC(Cys) with MTSL, continuous wave EPR spectra were detected at room temperature on an E500 Elexsys Bruker spectrometer equipped with a super high *Q* cavity. The samples were thawed on ice, and 20 μl were transferred into a 1.5-mm outer diameter glass capillary. A 14-mT field sweep was performed, with 0.15 mT modulation amplitude, 7.96 milliwatt incident microwave power, and ∼9.38 GHz frequency. As the measured signal is the first derivative, the resulting curve has to be integrated to obtain the absorbance spectrum. Double integration yields the spin concentration as horizontal asymptote. The area under the absorbance spectrum is proportional to the spin concentration. The correlation factor was experimentally determined with a solution of known concentration of tempol in water.

##### Distance Measurement Using DEER

In this work, the *Q*-band DEER experiments were performed as described in Polyhach *et al.* (56). By using deuterated cryoprotectants, the relaxation time can be increased thus increasing range of distance measurement and sensitivity. As protein samples are analyzed at 50 K after flash-freezing in liquid nitrogen, the equilibrium population was observed, thus yielding a distance distribution rather than a single distance. For DEER, the sample was thawed on ice, and 50 μl was transferred into a quartz tube (3 mm outer diameter, Aachener Quarz-Glas Technologie Heinrich) and flash-frozen in liquid nitrogen. Optimization and evaluation of *Q*-band DEER experiments are described in Bordignon and Polyhach (57). Data were acquired for 4–12 h. Normalized experimental data (*V*(*t*) = *V*(0)) are background corrected to obtain the DEER form factor (*F*(*t*) = *F*(0)) by division by the background function (which is mainly due to intermolecular interactions). The form factor oscillates around a horizontal line at (1 − Δ) (Δ is the modulation depth) after background correction. The distance distribution is extracted from the form factor using DeerAnalysis2013 (55).

##### Analysis of Interactions between MxiC and Other Proteins Using EPR

To analyze the interaction between MxiC and other proteins, we performed continuous wave X-band EPR spectroscopy at room temperature to detect eventual changes in the dynamic properties of the two spin labels on MxiC upon complex formation. Each EPR sample was prepared in gel filtration buffer (20 mm Tris-HCl, pH 7.5, 100 mm NaCl), and contained a final concentration of ∼75 μm MxiC and 10-fold more of the other proteins (∼750 μm).

## Author Contributions

A. D. R., E. B., R. B. S., I. M. A., and A. J. B. conceived and designed the experiments; A. D. R., E. B., S. M., D. K. S., X. L., M. P., I. M., R. B. S., and A. J. B. performed the experiments; A. D. R., E. B., I. M. A., R. B. S., and A. J. B. analyzed the data; X. L. and M. P. generated reagents and materials; and A. D. R., E. B., I. M. A., R. B. S., and A. J. B. contributed to the writing of the manuscript. All authors approved the final version of the manuscript.

## Supplementary Material

Supplemental Data

## Footnotes

5 The abbreviations used are:

T3SS: type III secretion system
T3S: type III secretion
TC: tip complex
CBD: chaperone-binding domain
IPTG: isopropyl 1-thio-β-d-galactopyranoside
CR: Congo red
PDB: Protein Data Bank
cw: continuous wave
MTSL: (1-oxyl-2,2,5,5-tetramethylpyrroline-3-methyl)methanethiosulfonate
DEER: double electron-electron resonance
ANOVA: analysis of variance
mT: millitesla.
