# Supplementary material for: Steps for Shigella Gatekeeper Protein MxiC Function in Hierarchical Type III Secretion Regulation
Source: J Biol Chem. 2016 Dec 14;292(5):1705–23. doi: 10.1074/jbc.M116.746826 (PMC5290946; doi:10.1074/jbc.M116.746826)
Supplement: Supplemental Data [file supp_292_5_1705__index.html]

Steps for Shigella Gatekeeper Protein MxiC Function in Hierarchical Type III Secretion Regulation — Genetic and Biophysical Study of Shigella T3SS Component MxiC — Supplemental Data 

# Steps for *Shigella* Gatekeeper Protein MxiC Function in Hierarchical Type III Secretion Regulation

## Supplemental Data

- Supplemental Data
