# Supplementary material for: Steps for Shigella Gatekeeper Protein MxiC Function in Hierarchical Type III Secretion Regulation
Source: J Biol Chem. 2016 Dec 14;292(5):1705–23. doi: 10.1074/jbc.M116.746826 (PMC5290946; doi:10.1074/jbc.M116.746826)

SUPPLEMENTAL INFORMATION

Steps for *Shigella* Gatekeeper Protein MxiC Function in Hierarchical Type III Secretion Regulation

A. Dorothea Roehrich, Enrica Bordignon, Selma Mode, Da-Kang Shen, Xia Liu, Maria Pain, Isabel Murillo, Isabel Martinez-Argudo, Richard B. Sessions and Ariel J. Blocker

This file contains:

Supplementary Figures S1-12

Supplementary Figure Legends

Supplementary Tables S1-S4

Supplementary References

SUPPLEMENTRAY FIGURES, LEGENDS AND TABLES

Figure S1. Structure of *Shigella* MxiC and its *Yersinia* homologs YopN & TyeA. (A) Top, MxiC structure 2VJ4 (chain A) coloured from blue at the N-terminus, where MxiC74 is the first residue in the first crystallised helix, to red at the C-terminus. Therefore, N-terminus and most of CBD are not shown. (B) Same MxiC rotated by 90° about its long axis. (C) Schematic showing MxiC's 3 α-helical X-bundles. (D) MxiC coloured in cyan & blue and (E) YopN (1XL3) coloured in green & TyeA in red to show the relation between their homologous domains/proteins.

Figure S2: Reduced secretion of MxiC mutant proteins has different causes. Several of the *mxiC* mutants are only poorly secreted. We wondered whether their secretion was deregulated or whether the mutant proteins were not "secretable". To test this, the relevant mutants were combined with *ΔipaB*. The *ΔipaB* mutant is a constitutive secreter that also constitutively secretes MxiC (1,2). If the MxiC mutant proteins are not secreted or secreted at much lower levels than the wild-type protein, then we can conclude that they have an intrinsic secretion defect, i.e. the mutation affects the "secretability" of the protein. If, however, they are now secreted at levels similar to the wild-type protein, then their secretion is solely deregulated. Expression levels of MxiC in whole culture lysates (*bottom panel*) were compared to the amount of MxiC secreted after CR induction (*top panel*). Samples from *Shigella* wild-type, *ΔipaB* and *ΔmxiC* or *ΔipaB ΔmxiC* deletion mutants containing plasmids expressing either wild-type *mxiC* or *mxiC* mutants were prepared as described in the Materials and Methods, Western-blotted with an antibody against MxiC. Cultures were grown with 25 µM IPTG except for the strains containing *mxiCΔNterm* where 100 µM IPTG was used. Results shown are representative of two independent experiments. We do not know why MxiC secretion in *ΔipaB* and *ΔipaB ΔmxiC*/*mxiC*+ is not equivalent but, all mutants should be compared to *ΔipaB ΔmxiC*/*mxiC*+.

Figure S3: Alignment of the C-terminus of MxiC. Alignment of the C-terminal helix of MxiC (helix 13) with its homologues. The alignment was cropped from Figure 8 of (3). Residues with more than 40% similarity are coloured: red for acidic, blue for basic, yellow for polar/uncharged and green for hydrophobic/nonpolar residues. "Sf_MxiC" is *S. flexneri* MxiC and Ec_SepL is EPEC SepL.

Figure S4: The extreme C-terminus of MxiC is critical for function. (A) Protein secretion in response to the artificial inducer Congo red (CR). *Shigella* wild-type, *ΔmxiC* mutant, complemented strain (*ΔmxiC*/*mxiC*+) and *mxiCΔCterm* (in the *ΔmxiC* background) were grown with 25 µM IPTG where required. Samples were collected as described in the Materials and Methods, Silver stained (*top panel*) and Western blotted with the indicated antibodies (*bottom panels*). **(B)** Exponential leakage. Samples were collected as described in the Experimental Procedures and Silver stained. **(C)** Total protein expression levels in whole culture lysates. Samples were collected as described in the Materials and Methods and Western-blotted with the indicated antibodies. Data shown are representative of two independent experiments.

Figure S5: The chaperone binding domains of MxiC homologues are conserved. Alignment of the N-terminal chaperone binding domains of MxiC homologues. The alignment was cropped from Figure 8 in (3). As the published alignment only starts at residue 43 of MxiC (Sf_MxiC), the sequences were extended to show the poorly aligned N-terminal part (tinted area) of the chaperone-binding domain of YopN (Ye_YopN). The disordered region of YopN and its regions contacting the chaperones SycN (green dots) and YscB (blue dots), respectively, are highlighted. Residues with more than 40% similarity are coloured: red for acidic, blue for basic, yellow for polar/uncharged and green for hydrophobic/nonpolar residues. Residues mutated in MxiC in our work are marked with arrows.

Figure S6: For different proteins, different percentages of the total protein pool are secreted. Wild-type *Shigella* was grown to exponential phase, resuspended in PBS to an OD600 of 15 and secretion was induced by addition of 200 µg/ml CR. After 8 min at 37° C, the amount of protein in the supernatant was compared to whole culture lysates by Western blotting. Intensities were quantified using Odyssey software (Li-Cor) and a linear fit of the dilution series was calculated. Results from two independent experiments are shown, error bars indicate standard deviations. Statistical analysis was performed using an ANOVA with a Tukey's post hoc test. There is an overall difference between proteins ($p < 0.01$) and in the pairwise comparisons, significant differences were observed between MxiC and IpaB or IpaC ($p < 0.01$ and 0.05, respectively) and between IpaD and IpaB ($p < 0.05$).

Figure S7: Secretion and expression of MxiC is unaffected after deletion of Class I chaperones. (A) and **(C)** CR secretion samples from wild-type *Shigella* and indicated deletion mutants were collected as described in Experimental Procedures, Silver-stained (top panel) and Western-blotted with an antibody against MxiC. All samples in the same panel were analysed on the same blot. **(B)** and **(D)** Total protein expression levels in whole culture lysates. Samples were collected as described in Materials and Methods section 2.7.1 and Western-blotted with an antibody against MxiC. Data shown for the double mutants are representative of two independent experiments. Data shown for the triple mutant are representative of three independent experiments, partly performed in duplicates/triplicates. Secretion and expression of MxiC was quantified on Western blots and normalised for a wild-type analysed on the same blot. Neither secretion ((90 -/+43)% of wild-type) nor expression ((93 -/+ 23)% of wild-type) of MxiC is significantly affected by the deletion of all three known Class I chaperones. As additional bands were observed in the *ΔipgE Δspa15* double deletion mutant, we analysed the proteins secreted in the *ΔipgE Δspa15* double mutant by mass spectrometry (4). As expected for a strain lacking *ipgE* and *spa15*, secretion of IpgD and several Osp proteins (e.g. OspC1, OspD2, Osp4) was reduced. We also found that secretion of several IpaH late effector proteins (IpaH4.5, IpaH9.8, IpaH7.8) was significantly increased. As these proteins have a molecular weight between 61 and 65 kDa, this could account for the additional bands observed in panel A. It is possible that reduced stability of the anti-activator OspD1 in the absence of its chaperone Spa15 liberates the transcriptional activator MxiE (5) leading to expression of IpaH late effector proteins.

Figure S8: MxiC was modelled to resemble the "bent" conformation of YopN/TyeA. (A) Crystal structure of YopN/TyeA (*top*), the bent MxiC model (*middle*) and the MxiC crystal structure (*bottom*). The YopN/TyeA crystal structure (PDB code 1XL3, chains A and C, (6)) is depicted in green (YopN) and red (TyeA). The broken helix that leads to a bend in the molecule is shown as yellow "pipe". MxiC (PDB code 2VJ4, chain A, Deane et al. (2008b)) is displayed in cyan (N-terminal region, residues 64 to 253) and blue (C-terminal region, residues 254 to 355). Helix 9 that is extended in the crystal structure (orange, bottom) and bent in the model (purple, middle) is displayed as "pipe". Residue V256 where a proline was introduced in MxiC is highlighted in red. **(B)** Alignment of the helix 9 of MxiC with its homologues. The alignment was cropped from Figure 8 in (3). Residues with more than 40% similarity are coloured, red for acidic, blue for basic, yellow for polar/uncharged and green for hydrophobic/nonpolar residues. "Sf_MxiC" is *S. flexneri* MxiC, "Ye_YopN" is *Y. enterocolitica* YopN. Helices observed in the protein crystals of MxiC (PDB code 2VJ4) and YopN (PDB code 1XL3) are displayed below the alignment. The secondary structure was predicted for the MxiC sequence using Quick2D (http://toolkit.tuebingen.mpg.de/quick2_d), a server combining multiple prediction methods: PSIPRED, JNET and Prof. The resulting predictions are displayed at the bottom. "H" stands for α-helix and "E" stands for β-sheet.

Figure S9: Predicted distance distributions for spin labels attached to Cys247 and Cys290 differ between the predicted extended and "bent" MxiC forms. (A) and **(C)** Predicted rotameric distribution of MTSL spin labels attached to Cys247 and Cys290 in

MxiC(Cys). The simulation was made using MMM software (7) and is based on the MxiC crystal structure (A, PDB code 2VJ4 chain A, (8)) and our "bent" MxiC model (Figure S6). The different rotamers of the spin labels are shown in yellow and the midpoints of the N-O bond are shown as red dots whose radii correspond to the rotamer population. The C-termini of MxiC are on the right side of the molecule. **(B)** and **(D)** Predicted distance distributions (P(r)) for the conformational models presented in **(A)** and **(C)**. The predicted major peak for extended MxiC **(C)** is close to 3 nm, while a peak close to 1 nm was predicted for the "bent" form **(D)**.

Figure S10: Introduction and removal of cysteines does not affect MxiC function. (A) Protein secretion in response to the artificial inducer Congo red (CR). *Shigella ΔmxiC* mutant, complemented strain (*ΔmxiC*/*mxiC*+) and *mxiC(Cys),* i.e. *mxiC(C184A/C233S/A247C/S290C),* mutant (in the *ΔmxiC* background) were grown with 25 μM IPTG for *ΔmxiC*/*mxiC* or 50 μM IPTG for *mxiC(Cys)*. Samples were collected as described in Experimental Procedures and Silver-stained. **(B)** Total protein expression levels in whole culture lysates. Samples were collected as described in Experimental Procedures and Western-blotted with an antibody against MxiC. Results shown are representative of two independent experiments.

Figure S11: Purification and labelling of proteins for EPR and DEER analysis. The success of the different purification protocols and steps is shown on the SDS-PA gel montages. All gels were stained with Coomassie blue. **(A)** Purification and MTSL labelling of MxiC(Cys), i.e. $MxiC_{C184A/C233S/A247C/S290C}$. The His-tagged protein was purified recombinantly from *E. coli* BL21 (DE3) carrying pDR104 *mxiC(C184A/C233S/A247C/S290C)*. **(B)** Purification of His-Spa15. The His-tagged protein was purified recombinantly from *E. coli* B834 (DE3) carrying pET-28b*spa15.* **(C)** The His-tagged protein was purified recombinantly from *E. coli* BL21 (DE3)pLysS carrying pET15b*his6-ipaDΔ15-332(C322S).* **(D)** Purification of IpgC/IpaC. The His-tagged protein complexes were purified recombinantly from *E. coli* BL21 (DE3) carrying pACYC*ipaC* and pET15bhis6-*ipgC.* F, number of eluted fraction. Those labelled in red were pooled, concentrated and used for the subsequent purification/labelling steps.

Figure S12. Comparison of the His-MxiC(Cys) experimental distance distributions with those simulated based on the seven different MxiC crystal structures. In the left column, V(t)=V(0) is the primary DEER data, in the right column, P(r) is the probability for the different distances. The red dotted lines show the simulation by MMM using the indicated chains from the crystal structures (8), the black lines show the experimental data for labelled His-MxiC(Cys).

Table S1: Strains used in this work

Strain name	Genotype	Reference
Shigella		
wild-type	wild-type M90T, serotype 5a	(9)
Δ*mxiC*	*mxiC*::*tetRA*	(2)
Δ*mxiC/mxiC*+	Δ*mxiC*/pIMA227; Note: this strain was used when no additional information is given	(2)
Δ*mxiC/mxiC*+ (pWSK29*)	Δ*mxiC*/pIMA221	This study
mxiCΔNterm	Δ*mxiC*/pDR60	This study
mxiCΔCterm	Δ*mxiC*/pDR73	This study
mxiCK66E	Δ*mxiC*/pDR96	This study
mxiCK68E	Δ*mxiC*/pDR100	This study
mxiC(M226K,L242D; hydrophobic)	Δ*mxiC*/pDR72	This study
mxiC(E201K,E276K,E293K; negative)	Δ*mxiC*/pDR67	(10)
mxiC(I251A,T253A,S254A, D255E; straight)	Δ*mxiC*/pDR93	This study
mxiC(T253G,S254G,D255G; wobble)	Δ*mxiC*/pDR92	This study
mxiCV256P	Δ*mxiC*/pDR91	This study
Δ*ipgE*	Δ*ipgE*::*kan*	This study
Δ*spa15*	Δ*spa15*::*kan*	This study
Δ*ipgA*	Δ*ipgA*::*kan*	This study
Δ*ipgE* Δ*spa15*	Δ*ipgE*::*FRT* Δ*spa15*::*kan* double mutant	This study

ΔipgE ΔipgA	*ΔipgE*::*FRT* *ΔipgA*::*kan* double mutant	This study
ΔipgE ΔipgA Δspa15	*ΔipgE*::*FRT* *ΔipgA*::*FRT* *Δspa15*::kan triple mutant	This study
ΔmxiC mxiH	ΔmxiCΔmxiH/ pUC18oc*mxiH*	This study
ΔmxiC mxiHK69A	*ΔmxiCΔmxiH*/ pUC18oc*mxiHK69A*	This study
mxiC mxiH	ΔmxiCΔmxiH/pIMA227 pUC18oc*mxiH*	This study
mxiC mxiHK69A	*ΔmxiCΔmxiH*/ pIMA227 pUC18oc*mxiHK69A*	This study
mxiCΔCBD mxiH	ΔmxiCΔmxiH/ pDR80 pUC18oc*mxiH*	This study
mxiCΔCBD mxiHK69A	*ΔmxiCΔmxiH*/pDR80 pUC18oc*mxiHK69A*	This study
mxiCK66E mxiH	ΔmxiCΔmxiH/pDR96 pUC18oc*mxiH*	This study
mxiCK66E mxiHK69A	*ΔmxiCΔmxiH*/pDR96 pUC18oc*mxiHK69A*	This study
mxiCK68E mxiH	ΔmxiCΔmxiH/pDR100 pUC18oc*mxiH*	This study
mxiCK68E mxiHK69A	*ΔmxiCΔmxiH*/pDR100 pUC18oc*mxiHK69A*	This study
mxiC(negative) mxiH	ΔmxiCΔmxiH/pDR67 pUC18oc*mxiH*	This study
mxiC(negative) mxiHK69A	*ΔmxiCΔmxiH*/pDR67 pUC18oc*mxiHK69A*	This study
mxiC(hydrophobic) mxiH	ΔmxiCΔmxiH/pDR72 pUC18oc*mxiH*	This study
mxiC(hydrophobic) mxiHK69A	*ΔmxiCΔmxiH*/ pDR72 pUC18oc*mxiHK69A*	This study
mxiCV256P mxiH	ΔmxiCΔmxiH/ pDR91 pUC18oc*mxiH*	This study
mxiCV256P mxiHK69A	*ΔmxiCΔmxiH*/ pDR91 pUC18oc*mxiHK69A*	This study

mxiCΔCterm mxiH	ΔmxiCΔmxiH/pDR73 pUC18oc*mxiH*	This study
mxiCΔCterm mxiHK69A	Δ*mxiC*Δ*mxiH*/pDR73 pUC18oc*mxiHK69A*	This study
Strain for purification of His6MxiC(Cys) for EPR	BL21(DE3)/pLysS/pDR104	This study
Strain for purification of His6IpaD(15-332 C322S) for EPR	*E.coli*BL21(DE3)/pLysS/pET15b*ipaD(15-332 C322S)*	This study
Strain for purification of His6IpgC and His6IpgC-IpaC for EPR	*E.coli* BL21 (DE3) Tuner/ pACYC*ipaC/* pET15b*ipgC*	(11)
Strain for purification of His6Spa15	*E.coli* B834 (DE3)/pET28b*spa15*	This study

Table S2. Plasmids used in this work

Plasmid	Description	Reference
pACT3	IPTG inducible plasmid, contains *lac* repressor, pACYC origin	(12)
pDR60	*mxiCΔNterm* (residues 2 to 30 removed) in pACT3, cloned via SacI/BamHI with own RBS	This study
pDR67	*mxiC(E201K,E276K,E293K)* in pACT3, cloned via SacI/BamHI with own RBS	(10)
pDR72	*mxiC(M226K,L242D)* in pACT3, cloned via SacI/BamHI with own RBS	This study
pDR73	*mxiCΔCterm* (residues 342 to 355 removed) in pACT3, cloned via SacI/BamHI with own RBS	This study
pDR80	*mxiCΔCBD* (residues 32 to 72 removed) in pACT3, cloned via SacI/BamHI with own RBS	This study
pDR91	*mxiCV256P* in pACT3, cloned via SacI/BamHI with own RBS	This study
pDR92	*mxiC(T253G,S254G,D255G)* in pACT3, cloned via SacI/BamHI with own RBS	This study
pDR93	*mxiC(I251A,T253A,S254A, D255E)* in pACT3, cloned via SacI/BamHI with own RBS	This study
pDR96	*mxiCK66E* in pACT3, cloned via SacI/BamHI with own RBS	This study
pDR100	*mxiCK68E* in pACT3, cloned via SacI/BamHI with own RBS	This study
pDR104	*mxiC(Cys) (C184A,C233S,A247C,S290C)* in pET28b (with N-terminal His-tag), cloned via NdeI/EcoRI	This study
pDR107	*mxiC*(Cys) *(C184A,C233S,A247C,S290C)* in pACT3, cloned via KpnI/SalI with own RBS	This study
pCP20	plasmid expressing FLP recombinase	(13)
pKD4	plasmid containing kanamycin resistance cassette flanked by FRT sites	(13)
pKD46	Plasmid expressing the λ Red recombinase	(13)
pET28b*mxiC*	*mxiC* in pET28b (with N-terminal His-tag), cloned via NdeI/EcoRI	(8)

pET28b*spa15*	full-length *spa15* in pET28b, cloned via NdeI/BamHI	(14)
pET15b*ipaD(15-332 C322S)*	*ipaD*, only residues 15 to 332 with C322S mutation in pET15b, cloned via NdeI and BamHI	This study
pACYC*ipaC*	*ipaC* in pACYC	(11)
pET15b*ipgC*	*ipgC* in PET15b	(11)
pEX-A-mxiC_EPR2	*mxiC*(C184A,C233S,A247C,S290C) in pEX-A cloning vector, custom	custom product from MWG Eurofins
pIMA208	*mxiC* in pUC19, cloned via SalI/BamHI	(2)
pIMA212	*mxiH* in pACT3, cloned via SacI/HindIII (Roehrich et al., 2013)	(10)
pIMA221	*mxiC* in pWSK29*, cloned via SacI/BamHI with own RBS	This study
pIMA227	*mxiC* in pACT3, cloned via KpnI/SalI with own RBS	(2)
pWSK29*	pWSK29* modified pWSK29 without T7 promoter; kind gift from Andrew Davidson (Bristol)	(15)
pUC18oc	pUC18 modified to carry constitutive operator sequence	(16)
pUC18oc *mxiH*	*mxiH* in pUC18 with modified operator, cloned via NdeI/PstI	This study
pUC18oc *mxiHK69A*	*mxiHK69A* in pUC18 with modified operator, cloned via NdeI/PstI	This study

Table S3: Primers used in this work

**Primer**	**Sequence**
mxiC_BamHI	CGC*GGATCC*CTGGATCACTTTTATCTCCTGTTATC
mxiC_341	CGC*GGATCC*TTA-TAGAATATTGATCGCAATTTCTCTTTCAC
mxiC_D32_72_F	GGAGATGAGACTGC-TGATAGTCAGGAACGTATTTTAG
mxiC_D32_72_R	CGTTCCTGACT-ATCAGCAGTCTCATCTCCATCATC
mxiC_EcoRI_R	GCGC*GAATTC*-TTATCTAGAAAGCTCTTTCTTGTATGCAC
mxiC_K66E_F	AAACAGAAGAGACCTTGAGgAACTGAAAGGAACAAATAGTG
mxiC_K66E_R	CACTATTTGTTCCTTTCAGTTcCTCAAGGTCTCTTCTG
mxiC_L242D_for	TCTGAGAAACCGAGCTGTAATGCTTATGAGTTTGGTTTTGTGgatTCTAAATTAATTGCAATTAAGATG
mxiC_M226K_rev	AAACTCATAAGCATTACAGCTCGGTTTCTCAGAATCttTGTCTACAATCAGTGACTGCTCTAC
mxiC_Ndel_F	GCGC*CA-TATG*CTTGATGTTAAAAATACAGGAG
mxiC_SacI	GCACGC*GAGCTC*AACTATAAAGTAGGTGATGTATGCTTG
mxiC_SacI_del30	GCACGC*GAGCTC*AACTATAAAGTAGGTGATGTATGGCTGATGCAGAGCTTGATTC
mxiC_SalI_rev	CTAG*GTCGAC*-TTATCTAGAAAGCTCTTTCTTGTATG
mxiC_straight_F2	TTGCAATTAAGATGgcTAGAgCTgCAGAaGTAATTTTTATGAAGAAACTG
mxiC_straight_R2	CATAAAAATTACtTCTGcAGcTCTAgcCATCTTAATTGCAATTAATTTAG
mxiC_wobble_F	TTGCAATTAAGATGATTAGAggTggAGgtGTAATTTTTATGAAGAAAC
mxiC_wobble_R	GTTTCTTCATAAAAATTACacCTccAccTCTAATCATCTTAATTGC
mxiC_V256P_F	GATGATTAGAACTTCAGACccAATTTTTATGAAGAAACTGGAATCC
mxiC_V256P_R	ATTCCAGTTTCTTCATAAAAATTggGTCTGAAGTTCTAATCATC
ipgA_KO_kanF	TCTCATTCTAATATATAGAAGGCCATAGAAATGTGTCGCAAACTATATGATGTGTAGGCTGGAGCTGCTTC
ipgA_KO_kanR	TTGTTTAGAATTTGCATGATACCCCCTATATGTTAGTTCACTTCTGAAGTCATATGAATATCCTCCTTAG
ipgE_KO_kanF	GGTGAAAGGGTATTCGTCATTTGTATAAGAGGAATATATGGAAGATT

	TAGGTGTAGGCTGGAGCTGCTTC
ipgE_KO_kanR	AATACGAAACGGGACATTAATACCCCTTCATTCTTCGCGCAAATTCA TCCCATATGAATATCCTCCTTAG
spa15_KO_kanF	AATACGAAACGGGACATTAATACCCCTTCATTCTTCGCGCAAATTCA TCCCATATGAATATCCTCCTTAG
spa15_KO_kanR	GAGCAATTTTGTATAGCTCATTGATTATAAGACCCCATTTAAGATTTC CACATATGAATATCCTCCTTAG
mxiH_NdeI_For	ATTA*CATATG*AGTGTTACAGTACCGAATG
mxiH_PstI_Rev	ATGC*CTGCAG*TTATCTGAAGTTTTGAATA
ipaD15_NdeI_For	AGTC*CATATG*TTCAGTCCAAACAATACCA
ipaD_BamHI_Rev	AGTC*GGATCC*TCAGAAATGGAGAAAAAGT

Start codons are shown in green, stop codons in red. Restriction enzyme sites are in italics. Mismatches in mutagenesis primers are indicated in lower case letters. In internal deletion primers the missing sequence is indicated by a dash. The pKD4 priming sites are shown in blue.

Table S4: Variables in protein purification conditions

	**Spa15**	**IpaD**	**IpgC/IpaC**	**MxiC** **C184A/C233S/A247C/S290C**
**Volume of culture**	1 L	1 L	1 L	2 L
**Volume of supernatant after sonication**	30 ml	25 ml	25 ml	60 ml
**Sonication**				
amplitude	60 %	50 %	60 %	60 %
repetitions	6	8	7	10
**Binding buffer**				
TrisHCl pH7.5	20 mM	20 mM	20 mM	20 mM
NaCl	150 mM	500 mM	500 mM	500 mM
Imidazole	15 mM	15 mM	10 mM	15 mM
**Elution buffer**				
TrisHCl pH7.5	20mM	20nM	20 mM	20mM
NaCl	150 mM	500mM	100 mM	100mM
Imidazole	1M	1M	1M	15mM
**Concentrator cut off (kDa)**	3	10	10	10

SUPPLEMENTARY REFERENCES

1. Menard, R., Sansonetti, P., and Parsot, C. (1994) The secretion of the Shigella flexneri Ipa invasins is activated by epithelial cells and controlled by IpaB and IpaD. *Embo J* **13**, 5293-5302
2. Martinez-Argudo, I., and Blocker, A. J. (2010) The Shigella T3SS needle transmits a signal for MxiC release, which controls secretion of effectors. *Mol Microbiol* **78**, 1365-1378
3. Pallen, M. J., Beatson, S. A., and Bailey, C. M. (2005) Bioinformatics analysis of the locus for enterocyte effacement provides novel insights into type-III secretion. *BMC Microbiol* **5**, 9
4. Roehrich, A. D. (2013) Regulation of type III secretion hierarchy in Shigella flexneri. *PhD thesis*
5. Parsot, C., Ageron, E., Penno, C., Mavris, M., Jamoussi, K., d'Hauteville, H., Sansonetti, P., and Demers, B. (2005) A secreted anti-activator, OspD1, and its chaperone, Spa15, are involved in the control of transcription by the type III secretion apparatus activity in Shigella flexneri. *Mol Microbiol* **56**, 1627-1635
6. Schubot, F. D., Jackson, M. W., Penrose, K. J., Cherry, S., Tropea, J. E., Plano, G. V., and Waugh, D. S. (2005) Three-dimensional structure of a macromolecular assembly that regulates type III secretion in Yersinia pestis. *J Mol Biol* **346**, 1147-1161
7. Polyhach, Y., Bordignon, E., and Jeschke, G. (2011) Rotamer libraries of spin labelled cysteines for protein studies. *Physical chemistry chemical physics : PCCP* **13**, 2356-2366
8. Deane, J. E., Roversi, P., King, C., Johnson, S., and Lea, S. M. (2008) Structures of the *Shigella flexneri* type 3 secretion system protein MxiC reveal conformational variability amongst homologues. *J Mol Biol* **377**, 985-992
9. Sansonetti, P. J., Kopecko, D. J., and Formal, S. B. (1982) Involvement of a plasmid in the invasive ability of Shigella flexneri. *Infect Immun* **35**, 852-860
10. Roehrich, A. D., Guillossou, E., Blocker, A. J., and Martinez-Argudo, I. (2013) Shigella IpaD has a dual role: signal transduction from the type III secretion system needle tip and intracellular secretion regulation. *Mol Microbiol* **87**, 690-706
11. Birket, S. E., Harrington, A. T., Espina, M., Smith, N. D., Terry, C. M., Darboe, N., Markham, A. P., Middaugh, C. R., Picking, W. L., and Picking, W. D. (2007) Preparation and characterization of translocator/chaperone complexes and their component proteins from Shigella flexneri. *Biochemistry* **46**, 8128-8137
12. Dykxhoorn, D. M., St Pierre, R., and Linn, T. (1996) A set of compatible tac promoter expression vectors. *Gene* **177**, 133-136
13. Datsenko, K. A., and Wanner, B. L. (2000) One-step inactivation of chromosomal genes in Escherichia coli K-12 using PCR products. *Proc Natl Acad Sci U S A* **97**, 6640-6645
14. Lillington, J. E., Lovett, J. E., Johnson, S., Roversi, P., Timmel, C. R., and Lea, S. M. (2011) Shigella flexneri Spa15 crystal structure verified in solution by double electron electron resonance. *J Mol Biol* **405**, 427-435
15. Wang, R. F., and Kushner, S. R. (1991) Construction of versatile low-copy-number vectors for cloning, sequencing and gene expression in Escherichia coli. *Gene* **100**, 195-199
16. Cheung, M., Shen, D. K., Makino, F., Kato, T., Roehrich, A. D., Martinez-Argudo, I., Walker, M. L., Murillo, I., Liu, X., Pain, M., Brown, J., Frazer, G., Mantell, J., Mina, P., Todd, T., Sessions, R. B., Namba, K., and Blocker, A. J. (2015) Three-dimensional electron microscopy reconstruction and cysteine-mediated crosslinking provide a model of the type III secretion system needle tip complex. *Mol Microbiol* **95**, 31-50

Figure S1

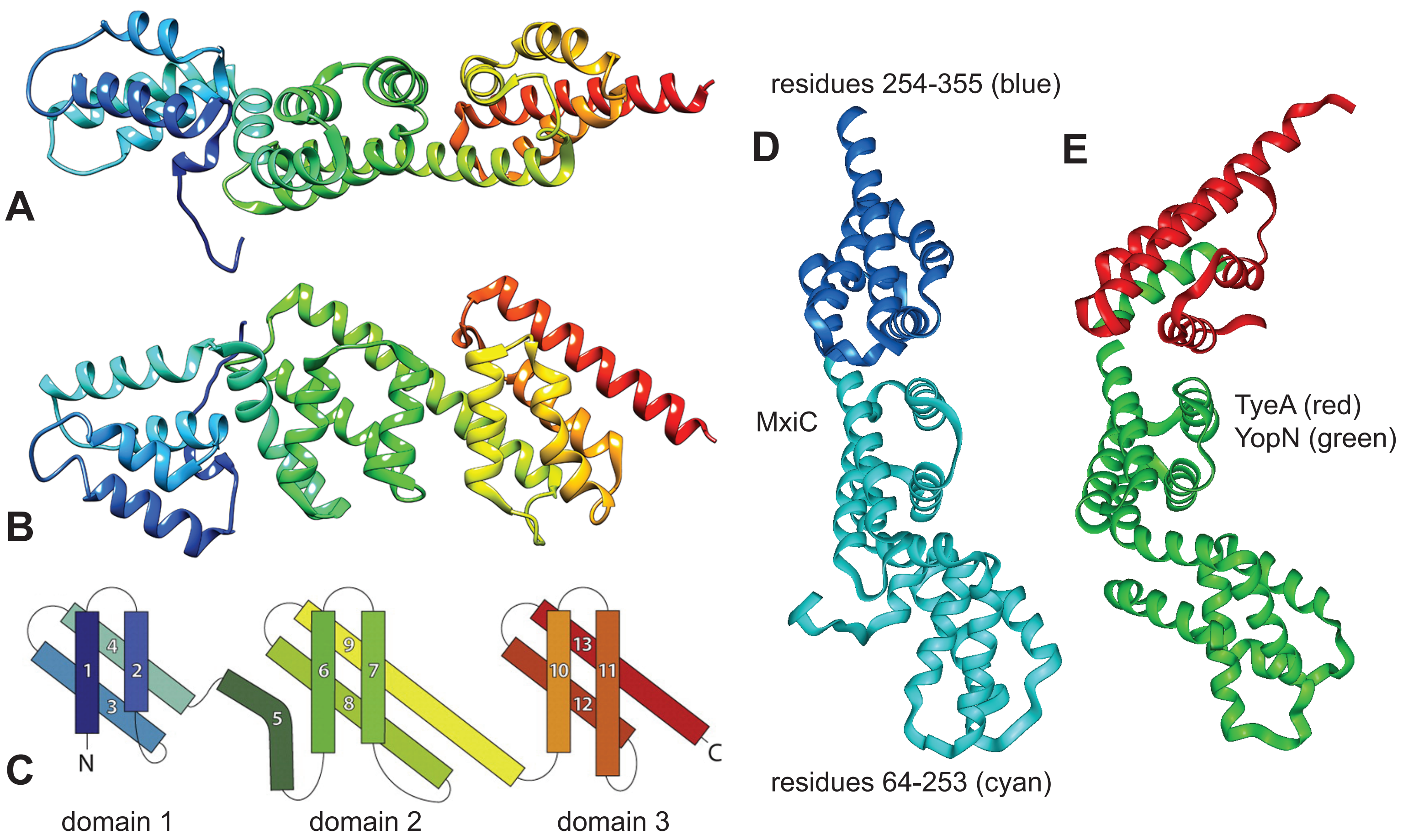
A
B
C
1
2
3
4
N
5
6
7
8
9
10
11
12
13
C
domain 1
domain 2
domain 3
D
residues 254-355 (blue)
MxiC
residues 64-253 (cyan)
E
TyeA (red)
YopN (green)

Figure S2

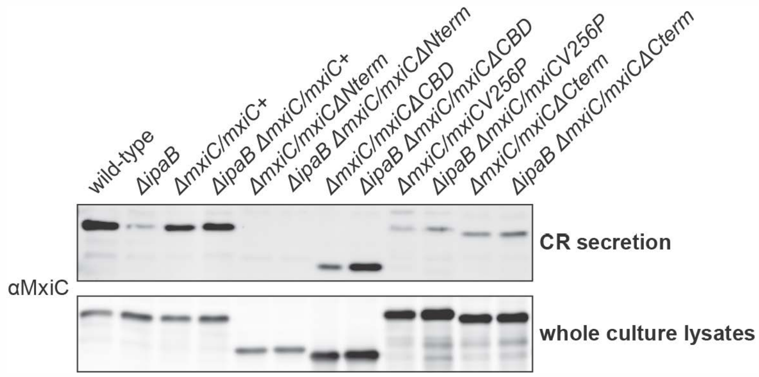

wild-type
ΔipaB
ΔmxiC/mxiC+
ΔipaB ΔmxiC/mxiC+
ΔmxiC/mxiCΔNterm
ΔipaB ΔmxiC/mxiCΔNterm
ΔmxiC/mxiCΔCBD
ΔipaB ΔmxiC/mxiCΔCBD
ΔmxiC/mxiCV256P
ΔipaB ΔmxiC/mxiCV256P
ΔmxiC/mxiCΔCterm
ΔipaB ΔmxiC/mxiCΔCterm
αMxiC
CR secretion
whole culture lysates

Figure S3

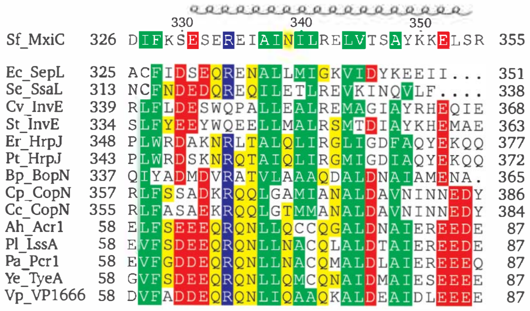
330 340 350
Sf_MxiC 326 DIFKSESEREIAINILRELVTSAYKKELSR 355
Ec_SepL 325 ACFIDSEQRENALLMIGKVIDYKEEII... 351
Se_SsaL 313 NCFNDEDQREQILETLREVKINQVLF.... 338
Cv_InvE 339 RLFLDESWQPALLEALREMAGIAYRHEQIE 368
St_InvE 334 SLFYEEYWQEELLMALRSMTDIAYKHEMAE 363
Er_HrpJ 348 PLWRDAKNRLTALQLIRGLIGDFAQYEKQQ 377
Pt_HrpJ 343 PLWRDSKNRQTAIQLIRGMIGDIAQYEKQQ 372
Bp_BopN 337 QIYADMDVRATVLAAAQDALDNAIAMENA. 365
Cp_CopN 357 RLFSSADKRQQLGAMIANALDAVNINNEDY 386
Cc_CopN 355 RLFASAEKRQQLGTMMANALDAVNINNEDY 384
Ah_Acr1 58 ELFSEEEQRQNLLQCCQGALDNAIEREEDE 87
Pl_LssA 58 EVFSDEEQRQNLLNACQLALDTAIEREEDE 87
Pa_Pcr1 58 EVFGDDEQRQNLLNACQMALDLAIEREEEQ 87
Ye_TyeA 58 GVFSDEEQRQNLLQMCQNAIDMAIESEEEE 87
Vp_VP1666 58 DVFADDEQRQNLIQAAQKALDEAIDLEEEE 87

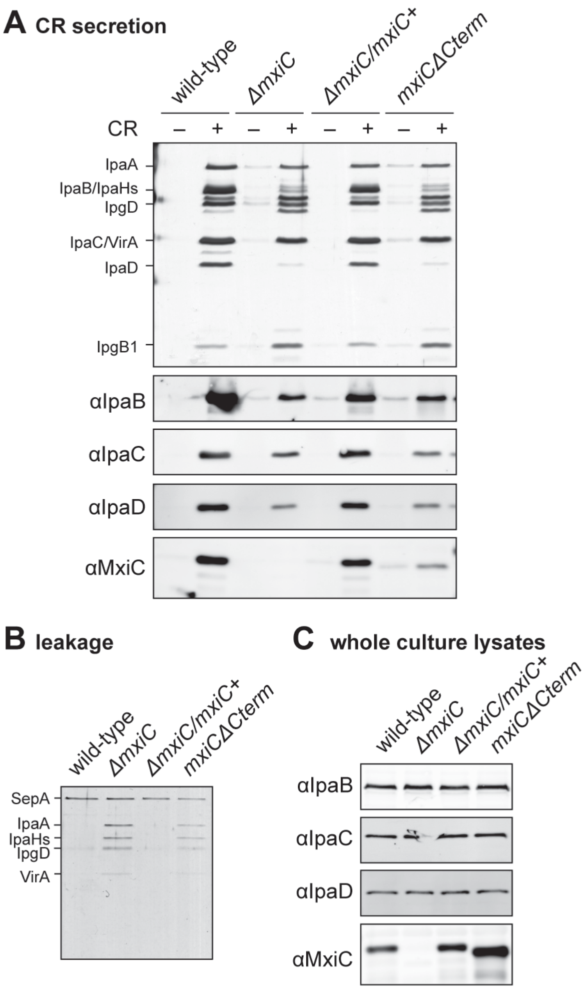
A CR secretion
wild-type
ΔmxiC
ΔmxiC/mxiC+
mxiCΔCterm
CR
IpaA
IpaB/IpaHs
IpgD
IpaC/VirA
IpaD
IpgB1
αIpaB
αIpaC
αIpaD
αMxiC
B leakage
wild-type
ΔmxiC
ΔmxiC/mxiC+
mxiCΔCterm
SepA
IpaA
IpaHs
IpgD
VirA
C whole culture lysates
wild-type
ΔmxiC
ΔmxiC/mxiC+
mxiCΔCterm
αIpaB
αIpaC
αIpaD
αMxiC

Figure S5

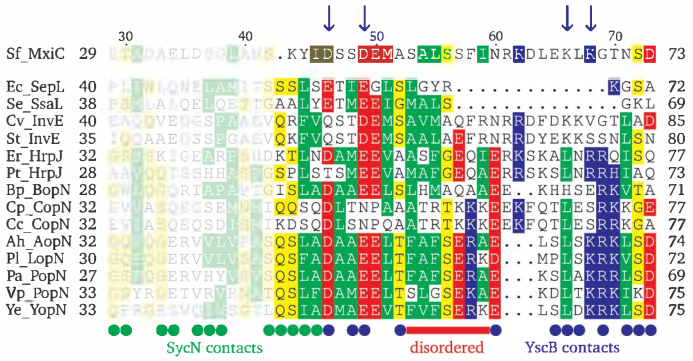
Sf_MxiC 29
73
30
40
50
60
70
Ec_SepL 40
Se_SsaL 38
Cv_InvE 40
St_InvE 35
Er_HrpJ 32
Pt_HrpJ 28
Bp_BopN 28
Cp_CopN 32
Cc_CopN 32
Ah_AopN 32
Pl_LopN 30
Pa_PopN 27
Vp_PopN 33
Ye_YopN 33
72
69
85
80
77
73
71
77
77
74
72
69
75
75
SycN contacts
disordered
YscB contacts

Figure S6

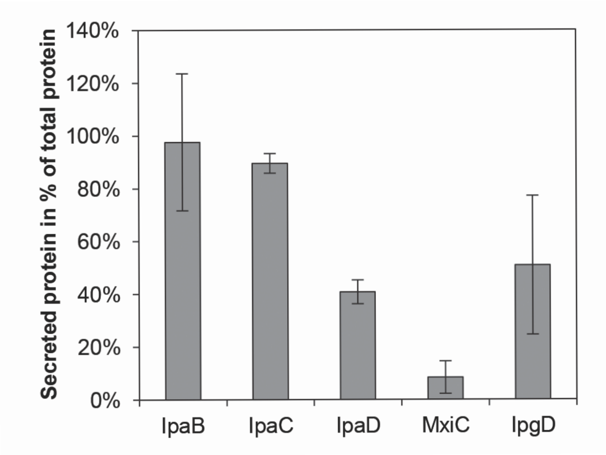

Secreted protein in % of total protein
140%
120%
100%
80%
60%
40%
20%
0%
IpaB
IpaC
IpaD
MxiC
IpgD

Figure S7

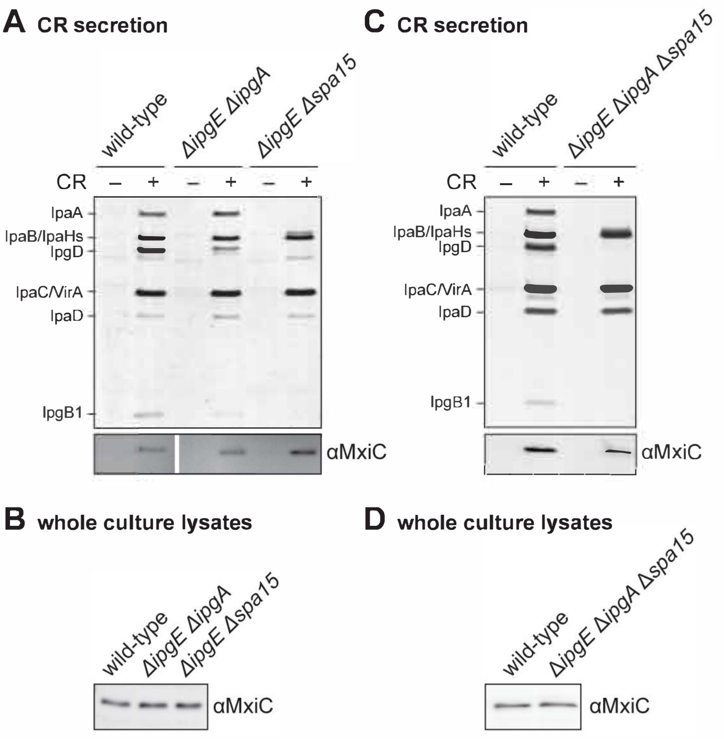
A CR secretion
wild-type
ΔipgE ΔipgA
ΔipgE Δspa15
CR
IpaA
IpaB/IpaHs
IpgD
IpaC/VirA
IpaD
IpgB1
αMxiC
B whole culture lysates
wild-type
ΔipgE ΔipgA
ΔipgE Δspa15
αMxiC
C CR secretion
wild-type
ΔipgE ΔipgA Δspa15
CR
IpaA
IpaB/IpaHs
IpgD
IpaC/VirA
IpaD
IpgB1
αMxiC
D whole culture lysates
wild-type
ΔipgE ΔipgA Δspa15
αMxiC

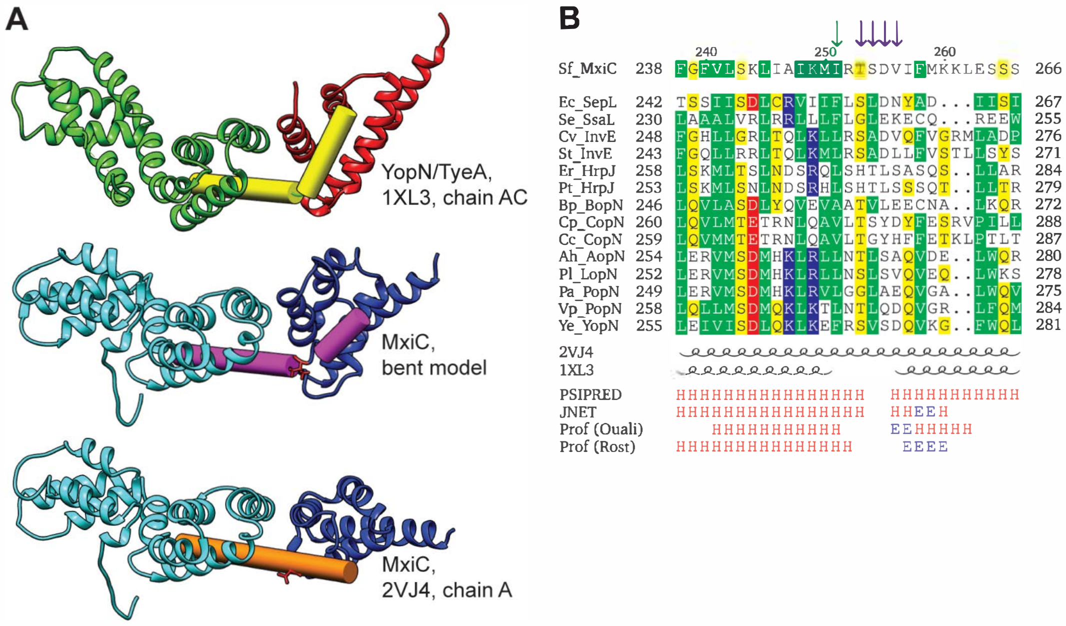
A
YopN/TyeA,
1XL3, chain AC
MxiC,
bent model
MxiC,
2VJ4, chain A
B
240
250
260
Sf_MxiC 238 FGFVLSKLIAIKMIRTSDVIFMKKLESSS 266
Ec_SepL 242 TSSIISDLCRVIIFLSLDNYAD...IISI 267
Se_SsaL 230 LAAALVRLRRLLLFLGLEKECQ...REEW 255
Cv_InvE 248 FGHLLGRLTQLKLLRSADVQFVGRMLADP 276
St_InvE 243 FGQLLRRLTQLKMLRSADLLFVSTLLSYS 271
Er_HrpJ 258 LSKMLTSLNDSRQLSHTLSASQS..LLAR 284
Pt_HrpJ 253 LSKMLSNLNDSRHLSHTLSSSQT..LLTR 279
Bp_BopN 246 LQVLASDLYQVEVAATVLEECNA..LKQR 272
Cp_CopN 260 LQVLMTETRNLQAVLTSYDYFESRVPILL 288
Cc_CopN 259 LQVMMTETRNLQAVLTGYHFFETKLPTLT 287
Ah_AopN 254 LERVMSDMHKLRLLNTLSAQVDE..LWQR 280
Pl_LopN 252 LERVMSDMHKLRLLNSLSVQVEQ..LWKS 278
Pa_PopN 249 LERVMSDMHKLRVLGGLAEQVGA..LWQV 275
Vp_PopN 258 LQLLMSDMQKLKTLNTLQDQVGR..LFQM 284
Ye_YopN 255 LEIVISDLQKLKEFRSVSDQVKG..FWQL 281
2VJ4
1XL3
PSIPRED HHHHHHHHHHHHHHHH HHHHHHHHHHH
JNET HHHHHHHHHHHHHHHH HHEEH
Prof (Ouali) HHHHHHHHHHH EEHHHHH
Prof (Rost) HHHHHHHHHHHHHHH EEEE

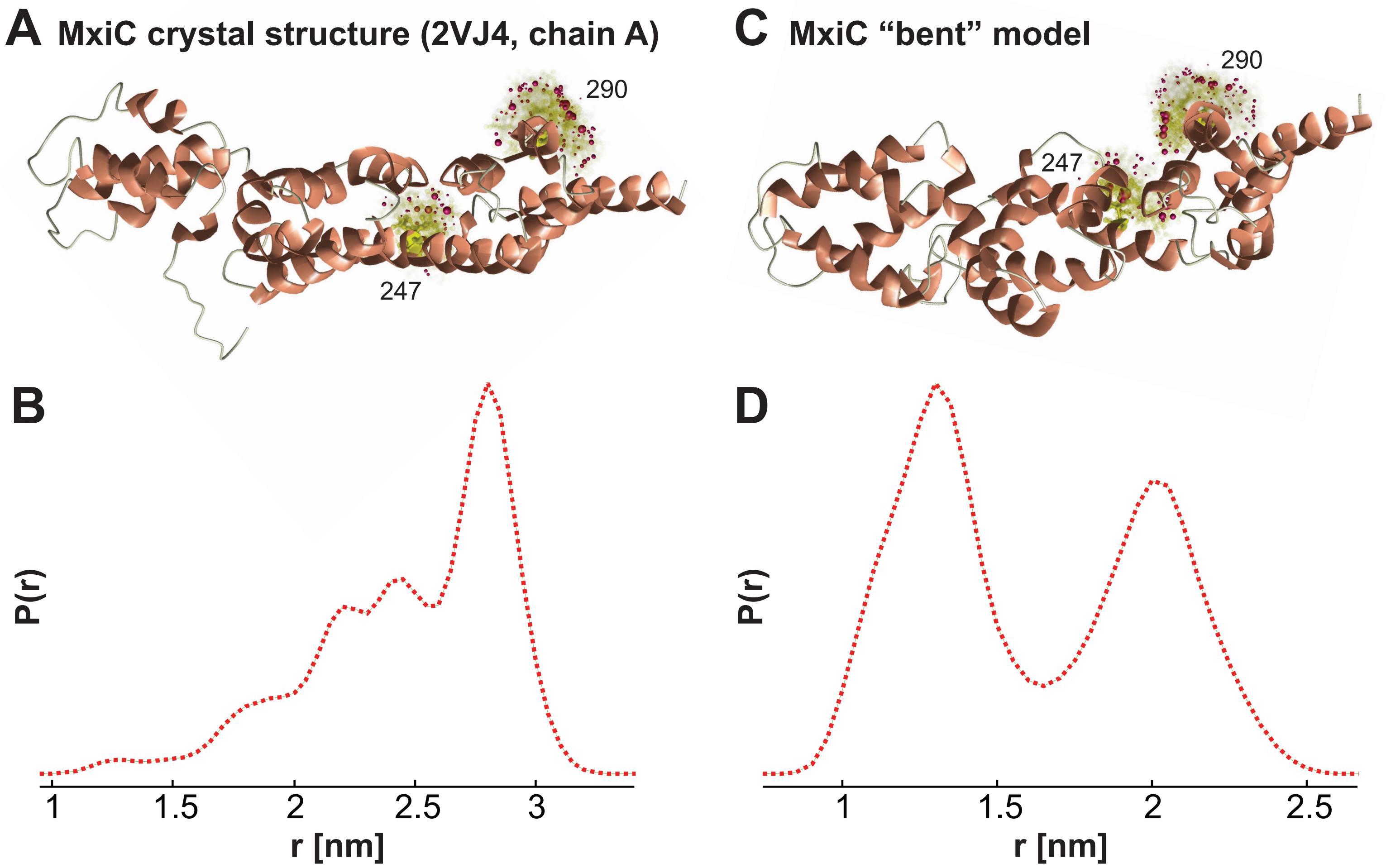
A MxiC crystal structure (2VJ4, chain A)
290
247
C MxiC "bent" model
290
247
B
P(r)
1
1.5
2
2.5
3
r [nm]
D
P(r)
1
1.5
2
2.5
r [nm]

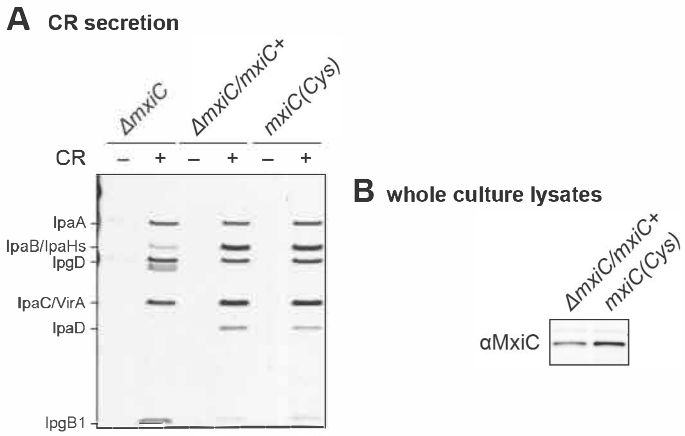
A
CR secretion
ΔmxiC
ΔmxiC/mxiC+
mxiC(Cys)
CR
–
+
–
+
–
+
IpaA
IpaB/IpaHs
IpgD
IpaC/VirA
IpaD
IpgB1
B
whole culture lysates
ΔmxiC/mxiC+
mxiC(Cys)
αMxiC

Figure S11

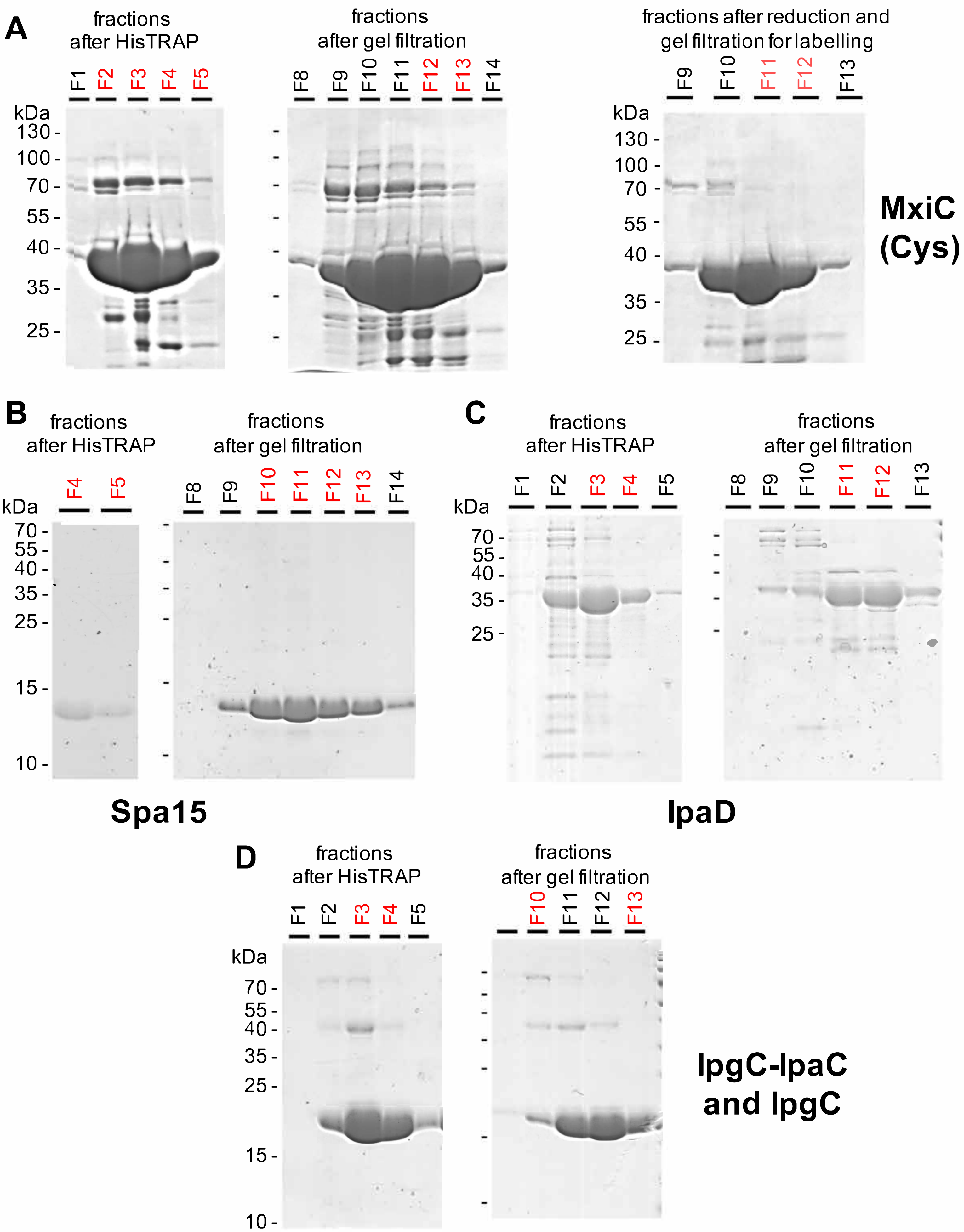
A
fractions after HisTRAP
F1 F2 F3 F4 F5
kDa
130 -
100 -
70 -
55 -
40 -
35 -
25 -
fractions after gel filtration
F8 F9 F10 F11 F12 F13 F14
fractions after reduction and gel filtration for labelling
F9 F10 F11 F12 F13
kDa
130 -
100 -
70 -
55 -
40 -
35 -
25 -
MxiC (Cys)
B
fractions after HisTRAP
F4 F5
kDa
70 -
55 -
40 -
35 -
25 -
15 -
10 -
fractions after gel filtration
F8 F9 F10 F11 F12 F13 F14
Spa15
C
fractions after HisTRAP
F1 F2 F3 F4 F5
kDa
70 -
55 -
40 -
35 -
25 -
fractions after gel filtration
F8 F9 F10 F11 F12 F13
IpaD
D
fractions after HisTRAP
F1 F2 F3 F4 F5
kDa
70 -
55 -
40 -
35 -
25 -
15 -
10 -
fractions after gel filtration
F10 F11 F12 F13
IpgC-IpaC and IpgC

Figure S12

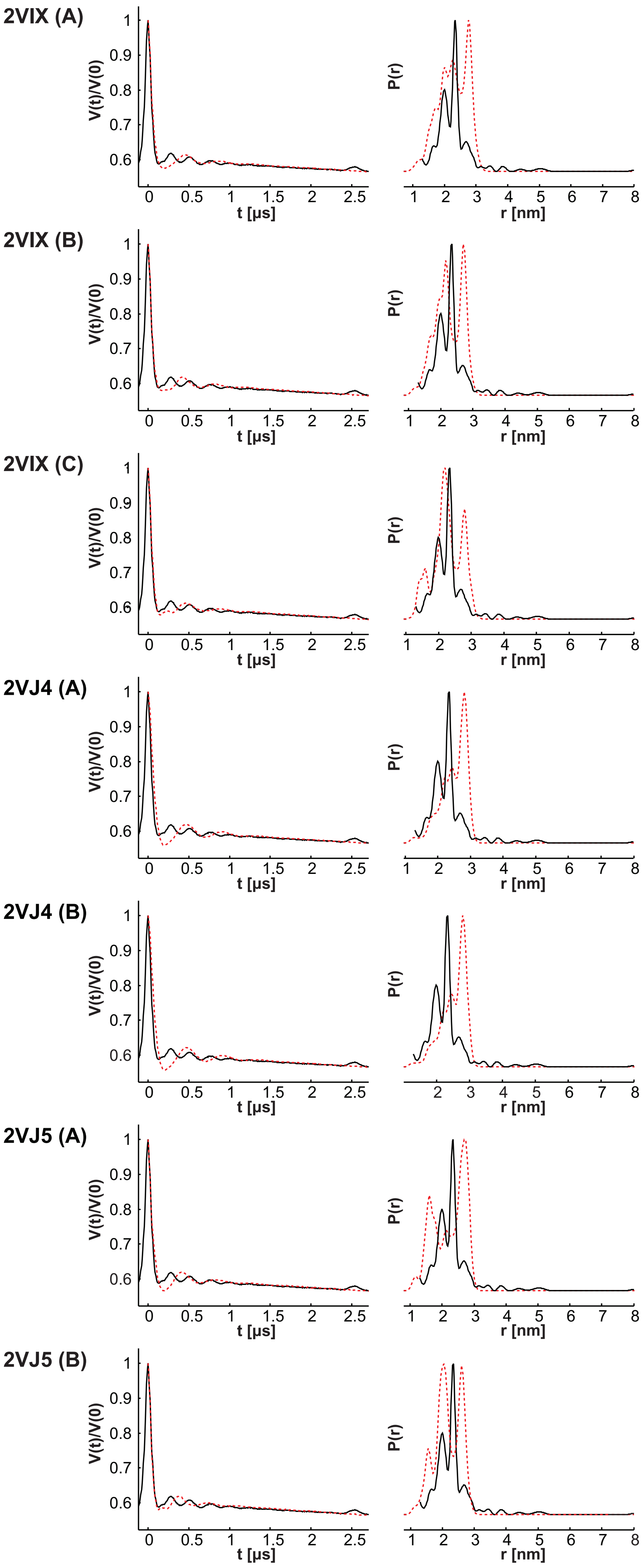

Supplement: Supplemental Data [file supp_M116.746826_suppdata.pdf]
